# Education and coronary heart disease: mendelian randomisation study

**DOI:** 10.1136/bmj.j3542

**Published:** 2017-11-13

**Authors:** Taavi Tillmann, Julien Vaucher, Aysu Okbay, Hynek Pikhart, Anne Peasey, Ruzena Kubinova, Andrzej Pajak, Abdonas Tamosiunas, Sofia Malyutina, Fernando Pires Hartwig, Krista Fischer, Giovanni Veronesi, Tom Palmer, Jack Bowden, George Davey Smith, Martin Bobak, Michael V Holmes

**Affiliations:** 1 Department of Epidemiology and Public Health, University College London, London, UK; 2 Department of Internal Medicine, Lausanne University Hospital, Lausanne, Switzerland; 3 Department of Complex Trait Genetics, Vrije Universiteit Amsterdam, Amsterdam, Netherlands; 4 Centre for Environmental Health Monitoring, National Institute of Public Health, Prague, Czech Republic; 5 Chair of Epidemiology and Population Studies, Institute of Public Health, Faculty of Health Sciences, Jagiellonian University Medical College, Krakow, Poland; 6 Institute of Cardiology, Lithuanian University of Health Sciences, Kaunas, Lithuania; 7 Research Institute of Internal and Preventive Medicine, Branch of the Institute of Cytology and Genetics, SB RAS, Novosibirsk, Russia; 8 Novosibirsk State Medical University, Novosibirsk, Russia; 9 Postgraduate Programme in Epidemiology, Federal University of Pelotas, Pelotas, Brazil; 10 Medical Research Council Integrative Epidemiology Unit at the University of Bristol, Bristol, UK; 11 Estonian Genome Center, University of Tartu, Tartu, Estonia; 12 Research Center in Epidemiology and Preventive Medicine, University of Insubria, Varese, Italy; 13 Department of Mathematics and Statistics, Lancaster University, Lancaster, UK; 14 School of Social and Community Medicine, University of Bristol, Bristol, UK; 15 Clinical Trial Service Unit and Epidemiological Studies Unit, Nuffield Department of Population Health, Big Data Institute, University of Oxford, Oxford, UK; 16 Medical Research Council Population Health Research Unit at the University of Oxford, Oxford, UK; 17 National Institute for Health Research Oxford Biomedical Research Centre, Oxford University Hospital, Oxford, UK

## Abstract

**Objective** To determine whether educational attainment is a causal risk factor in the development of coronary heart disease.

**Design** Mendelian randomisation study, using genetic data as proxies for education to minimise confounding.

**Setting** The main analysis used genetic data from two large consortia (CARDIoGRAMplusC4D and SSGAC), comprising 112 studies from predominantly high income countries. Findings from mendelian randomisation analyses were then compared against results from traditional observational studies (164 170 participants). Finally, genetic data from six additional consortia were analysed to investigate whether longer education can causally alter the common cardiovascular risk factors.

**Participants** The main analysis was of 543 733 men and women (from CARDIoGRAMplusC4D and SSGAC), predominantly of European origin.

**Exposure** A one standard deviation increase in the genetic predisposition towards higher education (3.6 years of additional schooling), measured by 162 genetic variants that have been previously associated with education.

**Main outcome measure** Combined fatal and non-fatal coronary heart disease (63 746 events in CARDIoGRAMplusC4D).

**Results** Genetic predisposition towards 3.6 years of additional education was associated with a one third lower risk of coronary heart disease (odds ratio 0.67, 95% confidence interval 0.59 to 0.77; P=3×10^−8^). This was comparable to findings from traditional observational studies (prevalence odds ratio 0.73, 0.68 to 0.78; incidence odds ratio 0.80, 0.76 to 0.83). Sensitivity analyses were consistent with a causal interpretation in which major bias from genetic pleiotropy was unlikely, although this remains an untestable possibility. Genetic predisposition towards longer education was additionally associated with less smoking, lower body mass index, and a favourable blood lipid profile.

**Conclusions** This mendelian randomisation study found support for the hypothesis that low education is a causal risk factor in the development of coronary heart disease. Potential mechanisms could include smoking, body mass index, and blood lipids. In conjunction with the results from studies with other designs, these findings suggest that increasing education may result in substantial health benefits.

## Introduction

Coronary heart disease (CHD) is the leading cause of death globally. Whereas the causal effects of risk factors such as smoking, high blood pressure, and raised low density lipoprotein cholesterol are generally accepted and reflected in disease prevention strategies, substantial uncertainty still surrounds other potential factors. Decades of observational studies have consistently associated socioeconomic factors such as higher education with decreased risk of CHD.^1^ ^2^ ^3^ ^4^ However, this association may not stem from an underlying causal effect but may arise owing to the methodological limitations of traditional observational research.^5^ ^6^ Clarifying whether the association between education and CHD is causal has widespread implications for our understanding of the causes of CHD, as well as for the potential development of novel population based approaches to its prevention. Unfortunately, randomised controlled trials are practically infeasible in this area, given the long (approximately 50 year) interval between exposure and outcome. Improving causal inference through other study designs is therefore necessary.

Mendelian randomisation analysis uses genetic variants associated with a risk factor (for example, education) to make causal inferences about how environmental changes to the same risk factor would alter the risk of disease (for example, CHD).^7^ Comparing the risk of disease across participants who have been grouped by their genotype enables the causal effect of a risk factor to be approximated with substantially less bias than in a traditional observational analysis. Genetic markers of a risk factor are largely independent of confounders that may otherwise cause bias, as genetic variants are randomly allocated before birth.^8^ This, as well as the non-modifiable nature of genetic variants, provides an analogy to trials, in which exposure is allocated randomly and is non-modifiable by subsequent disease.^8^

Until relatively recently, mendelian randomisation analyses have been conducted on single datasets in which data on genotype, risk factor, and outcome were measured for all participants (known as “one sample mendelian randomisation”). However, advanced analyses on pleiotropy require larger sample sizes to maintain statistical power. This would require data pooling across dozens of studies, which is administratively difficult to organise. As an alternative, summary level data from large genome-wide associations study (GWAS) consortia have become increasingly available in the public domain. Such data can be used to conduct mendelian randomisation analyses, whereby gene exposure measures are taken from one GWAS and gene outcome measures are taken from another GWAS (altogether known as “two sample mendelian randomisation”).^9^ Further methodological developments, including mendelian randomisation-Egger (commonly abbreviated to MR-Egger), weighted median mendelian randomisation, and mode based methods, can all be used as sensitivity analyses to additionally investigate any pleiotropic effects of the genetic variants (that is, when genetic variants for education exert their influence on heart disease through an “off-target” pathway that bypasses the education phenotype; see supplementary figure 1 for details.^9^ ^10^ ^11^ The mendelian randomisation method has successfully been applied to a range of biological and behavioural exposures.^12^ ^13^ We are aware of just two studies that have applied it to investigate a socioeconomic exposure: a polygenic score for education has previously been associated with the development of myopia and dementia.^14^ ^15^ However, these studies did not investigate the possibility of genetic pleiotropy.

Our primary research question was “Is there genetic support for the hypothesis that education is a causal risk factor in the development of CHD, and, if so, does education cause changes to conventional cardiovascular risk factors that could be mediators of this?” We firstly updated traditional observational estimates of the association between education and risk of CHD from several large studies and consortia. Secondly, we applied two sample mendelian randomisation analyses to investigate whether people with a genetic predisposition towards higher education have a lower risk of CHD. A recent GWAS from the Social Science Genetic Association Consortium (SSGAC) identified a large number of independent genetic variants (single nucleotide polymorphisms—SNPs) associated with educational attainment.^16^ We used 162 such SNPs to mimic the process of randomly allocating some participants to more education and other participants to less education. To compare the CHD risk of participants randomised in such a manner, we then used data from the Coronary Artery Disease Genome wide Replication and Meta-analysis plus the Coronary Artery Disease Genetics Consortium (CARDIoGRAMplusC4D) to see whether participants with genetic variants for longer education had an altered risk of CHD compared with participants with genetic variants for shorter education.^17^ Careful consideration of the results from such analyses, as well as the wider literature, can support inferences about the likely cardiac consequences from environmentally acquired alterations to education. We checked the robustness of our findings across a range of sensitivity analyses and additionally tested for reverse causation by checking whether those SNPs that best predict CHD also associate with educational outcomes. Supplementary figure 2 illustrates the mains steps taken in this study.

## Methods

Throughout all analyses, we defined education in the same way as in the original GWAS analysis, in which data from 65 studies were harmonised against the International Standard Classification of Education 1997 classification system (see supplementary table 1.3 of the original GWAS study^16^). After harmonisation, self reported educational attainment was modelled linearly, expressed as one standard deviation (that is, 3.6 years) of additional schooling. In this form, one year of vocational education was equivalent to one year of academic education, and we did not assume any qualitative differences in the type of education. We defined CHD as a composite of myocardial infarction, acute coronary syndrome, chronic stable angina or coronary stenosis of more than 50%, or coronary death.

### Observational association between education and CHD

In traditional observational analysis, we used a combination of cross sectional and prospective data, collected between 1983 and 2014 (table 1). For prevalent CHD cases in cross sectional data, we analysed 43 611 participants (1933 cases) from the National Health and Nutrition Examination Surveys (NHANES) (see supplementary figure 3).^26^ For incident CHD cases in prospective data, we analysed 23 511 participants (632 cases) from the Health, Alcohol and Psychosocial factors In Eastern Europe (HAPIEE) study^18^ and combined this with published estimates from 97 048 participants (6522 cases) of the Monica Risk, Genetics, Archiving and Monograph (MORGAM) study in Europe (see supplementary table 1 for case definitions and statistical details).^3^ ^19^

**Table 1 tbl1:** Details of studies and datasets included in analyses

Analysis/study	Risk factor/outcome	Participants (CHD cases)	Web source (if publicly available)
**Traditional observational analysis**			
NHANES	Years of education/non-fatal CHD	43 611 (1933)	www.cdc.gov/nchs/nhanes/
HAPIEE^18^	Years of education/fatal and non-fatal CHD	23 511 (632)	–
MORGAM^19^	Years of education/fatal and non-fatal CHD	97 048 (6522)	–
**Mendelian randomisation analysis (education to CHD and CHD to edication)**			
SSGAC^16^	Years of education	349 306	www.thessgac.org/data
CARDIoGRAMplusC4D^17^	CHD	194 427 (63 746)	www.cardiogramplusc4d.org/data-downloads/
**Mendelian randomisation analysis (education to conventional cardiovascular risk factors)**			
TAGC^20^	Smoking	74 053	www.med.unc.edu/pgc/results-and-downloads
ICBP^21^	Blood pressure	74 064	www.ncbi.nlm.nih.gov/projects/gap/cgi-bin/study.cgi?study_id=phs000585.v1.p1
GLGC^22^	LDL cholesterol, HDL cholesterol, and triglycerides	188 577	csg.sph.umich.edu/abecasis/public/lipids2013/
DIAGRAM^23^	Type 2 diabetes	149 821	diagram-consortium.org
MAGIC^24^	Glucose	133 010	www.magicinvestigators.org
GIANT^25^	Body mass index, height	339 224	portals.broadinstitute.org/collaboration/giant/

CARDIoGRAMplusC4D=Coronary Artery Disease Genome wide Replication and Meta-analysis (CARDIoGRAM) plus the Coronary Artery Disease (C4D) Genetics consortium; CHD=coronary heart disease; DIAGRAM=Diabetes Genetics Replication and Metaanalysis; GIANT=Genetic Investigation of Anthropometric Traits; GLGC=Global Lipids Genetic Consortium; HAPIEE=Health, Alcohol and Psychosocial factors In Eastern Europe; HDL=high density lipoprotein; ICBP=International Consortium for Blood Pressure; MAGIC=Meta-Analyses of Glucose and Insulin-related traits Consortium; LDL=low density lipoprotein; MORGAM=Monica Risk, Genetics, Archiving and Monograph; NHANES=National Health and Nutrition Examination Survey; SSGAC=Social Science Genetic Association Consortium; TAGC=Tobacco and Genetics Consortium.

### Genetic variants associated with education

We retrieved a shortlist of SNPs associated with educational attainment from a recent GWAS involving 405 072 people of European ancestry (table 1).^16^ For our main analysis, we used 162 independent SNPs associated (P<5.10^−8^; linkage disequilibrium *r^2^*<0.1) with education in a meta-analysis of the discovery (SSGAC) and replication (UK Biobank) datasets. Altogether, these 162 SNPs explained 1.8% of the variance in education. This is sufficient to generate a strong genetic instrument with which to derive unbiased causal estimates (see supplementary table 2 for power calculations). For our secondary analysis, we used another set of 72 independent SNPs (at *r* ^2^<0.1) that were associated with education in the discovery dataset (SSGAC) alone (293 723 participants; P<5.10^−8^) and that were subsequently found to be directionally consistent in an independent replication dataset (UK Biobank; see supplementary figure 4 for a summary of how SNPs were selected). We decided to use the larger set of instruments (with 162 SNPs) in our main analysis instead of the smaller set of instruments (with 72 SNPs) to maintain sufficient statistical power for our sensitivity analyses. To avoid potential biases that may arise when datasets contributing towards the SNP-to-exposure and SNP-to-outcome estimates overlap, we excluded studies in SSGAC that overlapped with CARDIoGRAMplusC4D (full details of these excluded studies are provided in supplementary methods 3.1). We then checked that the removal of these overlapping datasets from SSGAC had no material effect on the SNP-to-education estimates (see supplementary figures 5 and 6 for further details).

### Genetic variants associated with CHD

Data on CHD have been contributed by CARDIoGRAMplusC4D investigators and have been downloaded from www.cardiogramplusc4d.org. For each of the 162 SNPs associated with education, we retrieved summary level data for either the same SNP (115 of 162 SNPs) or for a proxy SNP in high linkage disequilibrium (47 of 162 SNPs at *r^2^*>0.8) from datasets totalling 63 746 CHD cases and 130 681 controls (see supplementary figure 7 for how the education SNPs were matched against the CHD GWAS dataset).^17^ We repeated a similar process for our secondary analysis using a set of 72 SNPs (supplementary figure 8).

### Statistical analyses

#### Traditional observational analyses

We used Cox proportional hazards and logistic regressions to calculate traditional observational estimates for incident and prevalent cases, respectively. Results were adjusted for age and sex. Further methodological details are given in supplementary methods 1.

#### Mendelian randomisation analyses

For all mendelian randomisation analyses, alleles from the SSGAC and CARDIoGRAMplusC4D datasets were aligned to correspond to an increase in educational attainment. To investigate whether education is likely to play a causal role in coronary heart disease, we used three mendelian randomisation approaches. Firstly, we used conventional (also termed ‘inverse variance weighted”) mendelian randomisation analyses, by regressing the SNP-education associations (exposure) against the SNP-CHD associations (outcome), with each SNP as one data point (details in supplementary methods 3.1**]**).

Secondly, we used three sensitivity analyses to investigate to what degree pleiotropic effects might bias the mendelian randomisation causal estimates. These methods allow some of the mendelian randomisation assumptions to be relaxed. For example, mendelian randomisation-Egger relies on the InSIDE assumption, which requires that the magnitude of any pleiotropic effects (from SNPs to CHD, which bypasses education) should not be correlated with the magnitude of the main effect (from SNP to education).^10^ Median based and mode based methods posit that when looking at lots of SNPs (some of which may have pleiotropic effects on CHD), these pleiotropic effects are likely to be comparatively heterogeneous in nature and hence less likely to converge on a common median/modal estimate. In contrast, valid SNPs with no pleiotropic effects are more likely to show more uniform and homogeneous effects (on education and thereafter CHD), which makes them more likely to cluster towards the median/modal point estimate.^9^ ^27^ These methods are fully described in supplementary methods 3.2. Consistency of results across a range of methods that make different assumptions about pleiotropy strengthens causal inference, whereas divergent results may indicate that genetic pleiotropy is biasing some of these results (described in supplementary figure 1).

Thirdly, to check whether genetic risk for coronary events might be a causal factor for educational attainment, we did mendelian randomisation in the opposite direction (bidirectional mendelian randomisation) using 53 SNPs associated with CHD (supplementary methods 3.2.4). Under conditions of massive pleiotropy, genetic risk of coronary events might also predict educational outcomes.

To investigate potential mechanisms from education to CHD, we applied conventional mendelian randomisation to investigate whether genetic predisposition towards longer education could lead to improvements in the established cardiovascular risk factors. In this analysis, we discarded 60 SNPs with missing data on one of the cardiovascular risk factors from the 162 SNP instrument and thus used a smaller set of 102 SNPs (details in supplementary methods 3.3 and supplementary figure 4).

### Patient involvement

Patients were not involved in the design or implementation of this study. There are no specific plans to disseminate the research findings to participants, but findings will be returned back to the original consortia, so that they can consider further dissemination.

## Results

### Observational analyses

On the basis of NHANES data, each additional 3.6 years of education (1 SD) was associated with 27% lower odds of prevalent CHD (odds ratio 0.73, 95% confidence interval 0.68 to 0.78; illustrated in figure 1). In prospective analyses, 3.6 years of additional education was associated with a 20% lower risk of incident CHD in the HAPIEE and MORGAM studies, with a pooled hazard ratio of 0.80 (0.76 to 0.83). Cohort specific results from MORGAM are additionally shown in supplementary figure 9.^18^ ^19^ These observational estimates were robust to sensitivity analyses accounting for different case definitions, age at first CHD event, and potential confounding by other measures of socioeconomic position (supplementary table 3). We also saw evidence for a dose-response relation between the amount of education and risk of CHD (supplementary figures 10 and 11).

**Figure f1:**
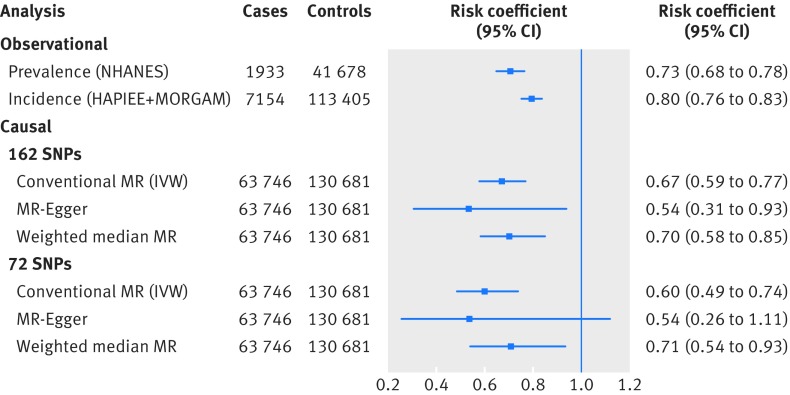
**Fig 1** Comparison of observational and causal estimates for risk of coronary heart disease (CHD), per 3.6 years of educational attainment. Two observational estimates are provided according to prevalent and incident CHD cases. Risk coefficient for observational incident cases was derived by meta-analysis of hazard ratios from Health, Alcohol and Psychosocial factors In Eastern Europe (HAPIEE) and Monica Risk, Genetics, Archiving and Monograph (MORGAM) studies. Risk coefficients for observational prevalent cases and six causal estimates from mendelian randomisation (MR) are all odds ratios (see supplementary methods for full description of each analysis). IVW=inverse variance weighted approach; NHANES=National Health and Nutrition Examination Survey

### Genetic association between education and CHD

After integrating two GWAS datasets and examining millions of SNPs across the entire genome, we found strong evidence for a negative genetic correlation between education and CHD (*r_g_*=−0.324; *r_g_^2^*=0.104; P=2.1×10^−12^; further details in supplementary methods 2).^28^ To interpret this, educational outcomes can vary as a result of genetic and non-genetic variance. Within the domain of genetic variance, approximately 10% of the genetic variance of education seems to be shared with the genetic variance of CHD, whereby this correlation is negative. This correlation can arise for various reasons, so we next did multiple mendelian randomisation analyses to investigate the presence and direction of any causal effects.

### Causal effect from education to CHD

Using conventional mendelian randomisation analysis, 1 SD longer education (due to genetic predisposition across 162 SNPs) was associated with a 33% lower risk of CHD (odds ratio 0.67, 0.59 to 0.77; P=3×10^−8^). Supplementary figure 12 additionally shows individual causal estimates from each of the 162 SNPs. As expected, sensitivity analyses using mendelian randomisation-Egger and weighted median mendelian randomisation provided less precise estimates than with conventional mendelian randomisation. Nonetheless, their causal estimates were similar in terms of direction and magnitude, and they were unlikely to have happened by chance alone (fig 1). We found little evidence of a non-zero intercept from the mendelian randomisation-Egger test (intercept β=0.004, −0.056 to 0.013; P=0.417), consistent with the hypothesis that genetic pleiotropy was not driving the result. The mendelian randomisation regression slopes are illustrated in supplementary figures 13 and 14. A secondary set of analyses using a set of 72 SNPs instead of 162 SNPs yielded consistent results in terms of direction and magnitude (fig 1).

Further sensitivity analyses, using both sets of instruments, are reported in supplementary table 4. Briefly, an analysis that can account for some measurement error in our genetic instruments for exposure (so-called mendelian randomisation-Egger+SIMEX) gave similar findings.^29^ Results from modal based mendelian randomisation approaches were consistent with the hypothesis that genetic pleiotropy was not driving the conventional mendelian randomisation result. We also did robustness checks by omitting SNPs with higher levels of missing data, as well as SNPs that were available in the CHD GWAS dataset in the form of a proxy SNP. These gave similar results in terms of direction, magnitude, and statistical significance. Collectively, all these sensitivity analyses make it less likely that the presence of pleiotropic effects, or missing data, grossly biased our main causal analysis.

### Causal effect from CHD to education

We found little evidence for the hypothesis that genetic liability for CHD risk is associated with educational outcomes. Namely, 1-log greater genetic risk of CHD was associated with 2.4 (−16.6 to 21.4) days of longer educational attainment. Results were unchanged after application of mendelian randomisation-Egger and weighted median mendelian randomisation (fig 2). The results from individual SNPs are shown in supplementary figures 18-20.

**Figure f2:**
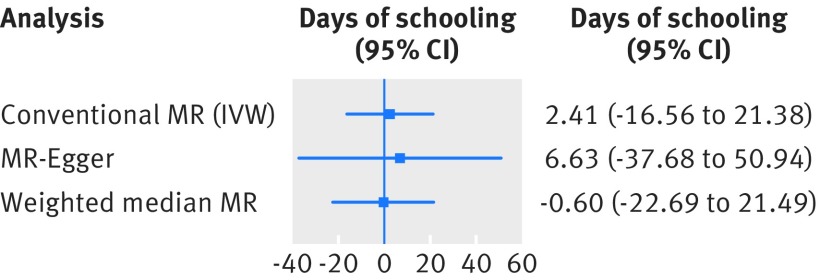
**Fig 2** Association of genetic liability to coronary heart disease (CHD) (exposure) on numbers of days of schooling (outcome). Causal estimates are expressed as difference in days of education per 1-log unit increase in risk of CHD as instrumented by 53 SNPs. Supplementary methods 3.2 details each mendelian randomisation (MR) analysis. IVW=inverse variance weighted approach

### Causal effect from education to cardiovascular risk factors

To identify potential risk factors that could mediate the association between education and CHD, we investigated whether genetic predisposition towards longer education was associated with established cardiovascular risk factors. Table 2 shows that, in conventional mendelian randomisation analyses, a 1 SD longer education (due to genetic predisposition across 102 SNPs) was associated with a 35% lower odds of smoking, 0.17 lower body mass index, 0.14 mmol/L lower triglycerides, and 0.15 mmol/L higher high density lipoprotein cholesterol, with a P value smaller than 0.001 for each of these four outcomes. Associations with diabetes and systolic blood pressure were in the anticipated direction, but these effects may have been due to chance or insufficient statistical power (P values 0.05 to 0.08).

**Table 2 tbl2:** Causal effects from 3.6 years of education to 10 cardiovascular risk factors

Outcome	Causal effect (95% CI)^*^	P value
**Binary traits**		
Smoking status	0.65 (0.54 to 0.79)	≤0.001
Diabetes mellitus, type 2	0.75 (0.56 to 1.01)	0.057
**Continuous traits**		
Systolic blood pressure	−1.36 (−2.85 to 0.12) mm Hg	0.075
Diastolic blood pressure	−0.23 (−1.22 to 0.76) mm Hg	0.645
Low density lipoprotein cholesterol	−0.03 (−0.10 to 0.05) mmol/L	0.513
High density lipoprotein cholesterol	0.15 (0.07 to 0.23) mmol/L	≤0.001
Triglycerides	−0.14 (−0.22 to −0.06) mmol/L	≤0.001
Glucose	−0.02 (−0.08 to 0.03) mmol/L	0.441
Body mass index	−0.17 (−0.26 to −0.08)	≤0.001
Height	0.06 (−0.03 to 0.16) cm	0.208

All analyses are based on a common set of 102 single nucleotide polymorphisms associated with education, available in eight genome-wide association study consortia (see supplementary methods 3.3).

*Estimates are expressed as absolute values for continuous risk factors and as odds ratios for binary traits; both correspond to 1 standard deviation longer education (equivalent to 3.6 years of schooling).

## Discussion

In this mendelian randomisation study, we found strong genetic support for the hypothesis that longer education has a causal effect on lowering the risk of coronary heart disease. Our findings using genetic data, which can be considered as “nature’s randomised trials,”^30^ were consistent with data from observational studies, and we found little evidence that our results may be driven by genetic pleiotropy. More specifically, 3.6 years of additional education (similar to an undergraduate university degree) is predicted to translate into about a one third reduction in the risk of CHD.

### Comparison with previous studies

A vast body of observational studies across a range of settings show an association between education and CHD. In contrast, comparatively few studies have explicitly investigated the causality of this association. The existing studies on causality come from three domains. Firstly, analyses of natural experiments have compared mortality before and after changes to compulsory schooling laws—for example, by looking at mortality rates in countries before and after the introduction of national legislation that increased minimum education. In the Netherlands, such changes were associated with reductions in all cause mortality.^31^ In the UK, the largest study so far reported causal effects on improving physical activity, body mass index, blood pressure, diabetes, CHD, and all cause mortality.^32^ An extension of this design is to compare geographical areas, such as the various states in the US. These studies initially suggested a large effect on all cause mortality, but this effect disappeared when state specific baseline trends were taken into account.^33^ ^34^ In Sweden, an intervention to extend compulsory schooling throughout a 13 year transition period in a stepped wedge design across multiple municipalities reported lower all cause mortality in those deaths occurring after age 40 (equivalent to hazard ratio of death of 0.86 (0.77 to 0.96) per 3.6 years of additional education).^35^

Another source of causal inference comes from studies on monozygotic twins. Within each pair, both twins are exposed to the same set of genetic exposures (and also some environmental exposures, called the “shared environment”). Consequently, any difference in disease outcome between twins cannot arise from genetic effects. If differences in outcome associate with differential exposure to non-shared features of the environment (such as one twin pursuing education longer than the other twin), and if the magnitude of this association is comparable to that seen in the general population, this makes less likely the possibility that the observational association is confounded by genetic (or shared environmental) factors. Although the twin method does not eliminate the possibility of confounding from other factors in the non-shared environment, it is a design with which to eliminate the possibility of confounding from genetic factors. Twin studies conducted in Denmark initially found evidence both for and against causal effects from education to mortality and CHD incidence.^36^ ^37^ The largest study to date from Sweden (which has twice the statistical power of the previous largest study) found strong evidence for causal effects.^38^ There, the association between years of education and lifespan did not attenuate at all when the conventional population based analysis was compared against the between twin analysis. Hence the twin literature suggests that, although only a handful of sufficiently powered studies exist, shared environmental factors (such as parenting) are not likely to cause substantial confounding. It also suggests that confounding from genetic factors (such as genetic differences in drive, motivation, personality, or innate intellect, all of which may predispose towards longer education) might not account for the observational associations between education and disease.

A parallel domain of research, using data from millions of non-identical siblings (that sometimes reached 100 times larger sample sizes than the twin studies), has also observed little attenuation of the association between education and subsequent mortality when comparing the general population analysis with the within sibling analysis.^39^ ^40^ As with twin studies, this also suggests that environmental and genetic factors shared by the siblings are unlikely to confound the observational association seen between education and disease. Although twin and sibling studies both leave open the possibility of confounding from non-shared environmental factors, taken together with our results (using an entirely different method), the wider body of evidence is more compatible with a causal interpretation, suggesting that increasing education may lead to a reduction in CHD.

Finally, some recent studies have also looked at specific genetic variants for education. An association was found between parental longevity and genetic markers for education in their offspring.^41^ However, causal directions and pleiotropy were not tested in this study. Others have used conventional mendelian randomisation and found that genetic variants for education predict myopia and dementia.^14^ However, these studies did not investigate pleiotropy of their genetic instruments. No mendelian randomisation studies of socioeconomic exposures have investigated any other disease outcome, such as cardiovascular diseases. Furthermore, most of the other designs listed above (including natural experiments and twin and sibling designs) have reported outcomes for all cause mortality. Few have reported cardiovascular mortality, and virtually none have reported fatal/non-fatal CHD, as we have.

### Strengths and limitations

Our study has important strengths. We investigated the causality of the association between an easily measured socioeconomic factor (education) and a common disease (coronary heart disease). We applied the mendelian randomisation design, which in conjunction with findings from other study designs should improve our understanding of causality by reducing bias from confounding. By integrating summary level data from more than half a million individuals, our study was well powered to derive robust causal effect estimates and also powered for multiple sensitivity analyses (which typically require larger sample sizes). We used recent state of the art methodological developments to thoroughly explore the possibility of pleiotropy in our genetic variants, for which we found little evidence.

Our study also has some limitations. Firstly, the genetic variants associated with education may instead mark more generic biological pathways (such as vascular supply or mitochondrial function), which could enhance systemic fitness, thereby leading to parallel increases in cognitive and cardiac function.^42^ ^43^ Under this scenario, which violates the InSIDE assumption, policy interventions to increase education may not translate into lower incidence of heart disease. However, such a scenario is less likely to lead to the consistent set of results we found across our sensitivity analyses, as this would require that pleiotropy occurs in a scenario in which the InSIDE assumption is violated (so that mendelian randomisation-Egger is biased), at least 50% of the information comes from SNPs with highly pleiotropic effects on heart disease, and these pleiotropic effects occurred in such a way as to make the causal estimates on heart disease seem very similar to one another. No definitive tests exist with which to verify such assumptions, meaning that triangulation of data from other sources and subjective judgment are needed to evaluate the plausibility of gross pleiotropic bias.^44^ We believe such pleiotropy to be unlikely for four reasons. Firstly, the effects from genetic pleiotropy would have to coincide with the non-genetic associations observed in studies of monozygotic twins; secondly, they would also have to coincide with the non-genetic associations observed in natural experiments. Thirdly, if education and CHD share some of their underlying genome-wide genetic architecture (as seen in our LD score regression), and if most of the top hits for education are strongly pleiotropic for CHD, then one might imagine the top hits for CHD to also pick up some of these pleiotropic traits. However, our reverse direction mendelian randomisation found a null estimate. Fourthly, despite gaps in our understanding of the biological mechanisms through which these 162 SNPs influence education, they are disproportionately found in genomic regions that regulate brain development, they are enriched for biological pathways involved in neural development, and they are preferentially expressed in neural tissue.^16^ As these 162 SNPs do not seem to have any expression or enrichment in cardiovascular tissues, this further narrows the scope for pleiotropy: any potential pleiotropy might have to exert a large effect on CHD via predominantly neurological pathways (for example, behaviours associated with obesity), rather than via global or systemic measures of fitness (such as mitochondrial function). Therefore, on balance, we believe that the scenario in which gross pleiotropy invalidates our sensitivity analysis is less consistent with the broader body of evidence, in comparison with the scenario in which our sensitivity analyses are valid. If our main and sensitivity analyses are valid, then policy interventions that mirror prolonged exposure to education (as indexed by our genetic instruments) should, on balance, probably prevent heart disease.

A second limitation is that to arrive at such a policy recommendation one would have to assume that genetic predisposition towards higher educational attainment causes the same behavioural and physiological consequences as environmentally acquired changes to educational attainment, such as from a policy intervention. It may be, however, that a year of additional education from genetic causes could trigger a different set of biological and behavioural mechanisms compared with a year of additional education resulting from policy change. We know very little about the mechanisms of these genetic effects. In the analyses we did in this study, we found some initial evidence that some of these genetic effects may be mediated via common cardiovascular factors such as smoking, body mass index, and lipids. In keeping with this, policy changes to education in the US and UK have also estimated some causal effects on smoking, body mass index, blood pressure, and diabetes,^32^ ^45^ which are broadly consistent with our findings. Few studies have measured the causal effects of policy interventions on blood lipids. Although a randomised controlled trial of education is difficult for CHD outcomes, owing to approximately 50 years of lag, future research using real life interventions may be able to measure effects on potential mediators, as these occur much sooner. A second response to this overall limitation is the analogy to other exposures (such as low density lipoprotein cholesterol and systolic blood pressure), for which genetic effects have mirrored findings from environmentally acquired changes (such as from randomised controlled trials of drug therapies.^46^ ^47^). Taken together, although our study makes no direct inference on what health effects may stem from a policy intervention that successfully increases education, we are cautiously optimistic that such a policy should lead to reductions in heart disease.

As a third limitation, we assumed the absence of dynastic effects, an assumption that is broken when parental genes associate with parental behaviours that directly cause a health outcome in the child.^48^ For example, parents with a genetic predisposition towards higher education may choose to feed their children a better diet. However, parental educational attainment has been shown to be a poor predictor of conventional cardiovascular risk factors in children.^49^ Fourthly, our observational and genetic data originate predominantly from samples of European origin in high income countries. We are thus unable to generalise these estimates to other populations, particularly to low income countries where cardiovascular diseases are less common. However, it may well be expected that socioeconomic factors mirror the pattern seen for other cardiovascular risk factors, whereby similar effects are typically seen across the world. For example, in the INTERHEART study, regional heterogeneity in the magnitude of associations was just as large for some conventional cardiovascular risk factors (eg, hypertension I^2^=85%, obesity I^2^=92%),^50^ as it was for some psychosocial risk factors (eg, depression I^2^=85%, general stress I^2^=79%).^51^ Fifthly, we do not know whether increasing education for the people with the least education will be as cardioprotective as increasing education for those with above average education. Nonetheless, a scenario of dose-response across the broad educational gradient is compatible with, firstly, the linear relation seen in the observational data. Secondly, it is also compatible with the concordance of findings from our study (which measures the average effect across the entire population) alongside the findings from studies of raising the school leaving age (which measure the effect among those with least education only).

### Potential mechanisms

The mechanisms that might mediate the association between education and CHD remain relatively unknown. Traditional observational associations have estimated that the association between education and CHD attenuates by around 30-45% after statistical adjustment for health behaviours and conventional cardiovascular risk factors (including smoking, blood pressure, and cholesterol); however, measurement error in such analyses can underestimate their mediating effect. This suggests that these factors could account for perhaps half of the association between education and CHD.^2^ ^52^ Our study found genetic predisposition towards longer education to associate with improved smoking, body mass index, and blood lipid profiles (with some borderline results for blood pressure and risk of diabetes). The degree of mediation should now be formally assessed with more extensive methods—for example, by applying two step mendelian randomisation.^53^ ^54^ If conventional risk factors do not completely account for the mechanism between education and CHD, then additional mechanistic hypotheses for investigations are needed. These could include education leading to improved use of healthcare services (from better health knowledge or fewer financial barriers to accessing care) or better job prospects, income, material conditions, social ranking and/or diet, all factors associated with education and CHD, many of which might be amenable to intervention.^4^

### What our study adds

After exposure to a socioeconomic factor, there is often a long latency period before the occurrence of common diseases (in this example, around 50 years). Consequently, this line of research is not particularly amenable to randomised controlled trials, which would otherwise settle questions of causality. This does not mean that these associations are less worthy of investigation, particularly as large point estimates open up the possibility of potentially large public health gains. The solution is to triangulate evidence from multiple study designs, each with its own strengths and weaknesses. The limited studies to date have suggested that a causal effect between socioeconomic exposures and all cause mortality is more likely than not to exist. Our study adds to this evidence by using an entirely new technique, which also suggests that a causal effect is more likely than not to exist between education and CHD.

### Implications for researchers

The main question for future research is “What mechanisms account for the strong association seen between genetic predisposition towards longer education and substantially lower risk of CHD?” Were it to be found that a health behaviour (such as diet) is an important mediator, then interventions on diet could become the cornerstone of policies designed to reduce health inequalities.

More molecular research is needed to delineate the mechanism, pleiotropic or not, through which these 162 education SNPs associate with cardiac outcomes. This could elucidate new causal mechanisms for CHD which, in turn, could lead to insights for potential drug discovery.

### Implications for clinicians and policymakers

Although uncertainty remains around the precise function of each of the 162 SNPs, their degree of pleiotropy with cardiac traits, and the mechanisms by which these genetic variants exert their cardioprotective influence, conclusions can still be drawn from the current body of evidence. Firstly, policies that increase education probably lead to non-health benefits, such as increased economic productivity, higher voter turnout, better governance, and improved life satisfaction.^55^ ^56^ Secondly, very little evidence exists to suggest that increasing education might subsequently harm health or wellbeing. Thirdly, although rigorous scientific debate needs to continue on the health consequences of increasing education, the current balance of opinion seems to weigh towards the side on which increasing education will probably improve a range of health outcomes (either to a smaller or larger degree). Little discussion has taken place about how to increase education in a manner that is practical, acceptable, affordable, and sustainable. Although our data make no claims on this, we note that interventions should be accompanied by careful monitoring for unforeseen side effects, especially in those people who may not thrive when forced into extended educational settings, which may otherwise aggravate health inequalities. To briefly begin this discussion, one can imagine a range of policies by analogy to how clinicians, public health practitioners, and policymakers encourage patients to stop smoking: by raising awareness (for example, mass marketing campaigns, personalised letters, or individual counselling), convenience of access (for example, changing the geographical dispersion of educational establishments or opportunities for flexible education), and/or finance (for example, tuition fees, accommodation costs, or stipends). One can also consider complementing some of these population level policies with individual level interventions (for example, advising adolescents on whether to pursue higher education).

### Conclusion

Our mendelian randomisation analyses found genetic support for the hypothesis that longer education plays a causal role in lowering the risk of coronary heart disease. Although completely ruling out possible pleiotropic effects is difficult, the sensitivity tests available to us gave little evidence that these could have driven our findings. In conjunction with the results from other study designs, increasing education is likely to lead to health benefits.

**What is already known on this topic**

Many observational studies have found that people who spend more time in educational settings subsequently develop less coronary heart disease
However, whether this association is causal is not clear, partly because randomised controlled trials are practically infeasible in this area
Few studies have applied mendelian randomisation to investigate how exposure to socioeconomic risk factors might causally change the risk of disease occurrence
No such study has done sensitivity analyses around genetic pleiotropy

**What this study adds**

Increasing the number of years that people spend in the educational system may lower their risk of subsequently developing coronary heart disease by a substantial degree
These findings should stimulate policy discussions about increasing educational attainment in the general population to improve population health

## Web Extra

Extra material supplied by the author

Supplementary materials

Analytical dataset

## Notes

We are grateful to the eight GWAS consortia (especially SSGAC and CARDIoGRAMplusC4D) for publicly sharing the genetic data we used in our causal analysis, to Reedik Mägi for assistance on LD score regression, and to Daniel J Benjamin for comments on an earlier draft of this article.

Contributors: TT and JV contributed equally to this paper and are joint first authors. TT had the idea for the study. AO obtained the genetic data. HP, AP, RK, AP, AT, SM, and KF obtained the observational data. MVH, GDS, JB, FPH, TP, and GV developed the study methods. JV, TT, FPH, and MVH did the analysis. All authors were involved in interpreting the data. TT, JV, and MVH wrote the first draft of the manuscript, and all authors critically revised it. TT, JV, and MVH are the guarantors.

Ethical approval: Participants gave informed consent for data sharing, as described in each of the discovery genome-wide association studies. Additional ethical approval was not needed for this study.

Transparency: The lead authors affirm that this manuscript is an honest, accurate, and transparent account of the study being reported; that no important aspects of the study have been omitted; and that any discrepancies from the study as planned (and, if relevant, registered) have been explained.

Data sharing: All of the summary level data used are available for instant download at the public repositories listed in table 1. The statistical code is available from the corresponding authors at t.tillmann@ucl.ac.uk and julien.vaucher@chuv.ch.
