# Supplementary material for: Education and coronary heart disease: mendelian randomisation study
Source: BMJ. 2017 Nov 13;358:j3542. doi: 10.1136/bmj.j3542 (PMC5594424; doi:10.1136/bmj.j3542)
Supplement: Supplementary file 1 — Supplementary materials [file tilt037504.ww1.pdf]

**Supplementary Appendix for**

# *Education and coronary heart disease: mendelian randomisation study*

# Table of contents


**Page**

**Supplementary methods** ........ 3-5

**Supplementary Tables**

**Table 1** Description of observational studies ........ 6
**Table 2** Power for conventional Mendelian randomization analysis ........ 7
**Table 3** Sensitivity analyses of observational estimates ........ 8
**Table 4** Sensitivity analyses of Mendelian randomization estimates ........ 9

**Supplementary Figures**

***Overview***

**Figure 1** Theoretical illustration of pleiotropic phenomena ........ 10
**Figure 2** Overview of the main steps in this study ........ 11

***Methods***

**Figure 3** Flowchart of participants in observational NHANES analysis........ 12
**Figure 4** Flowchart of 3 genetic instruments ........ 13
**Figure 5** Scatter plot comparing complete dataset vs. non-overlapping dataset (n=162 SNPs) ........ 14
**Figure 6** Scatter plot comparing complete dataset vs. non-overlapping dataset (n=72 SNPs) ........ 15
**Figure 7** Flowchart illustrating how SNPs identified in the SSGAC education GWAS were mapped against SNPs listed in CARDIoGRAMplusC4D (n=162 SNPs) ........ 16
**Figure 8** Flowchart illustrating how SNPs identified by SSGAC education GWAS were mapped against SNPs listed in CARDIoGRAMplusC4D (n=72 SNPs) ........ 17

***Observational Results***

**Figure 9** Observational estimates from MORGAM ........ 18
**Figure 10** Dose response, using observational NHANES data ........ 19
**Figure 11** Dose response, using observational HAPIEE data ........ 20

***Causal Results***

**Figure 12** Scatter plot of 162 SNPs associated with education, and their risk of CHD........ 21
**Figure 13** Scatter plot of 162 SNPs associated with education, and their risk of CHD (with MR-Egger)........ 22
**Figure 14** Funnel plot of 162 SNPS, showing education instrument strength against causal estimate on CHD. 23
**Figure 15** Scatter plot of the 72 SNPs associated with education and their risk of CHD ........ 24
**Figure 16** Scatter plot of the 72 SNPs associated with education and their risk of CHD (with MR-Egger) ........ 25
**Figure 17** Funnel plot of 72 SNPs, showing instrument strength against causal estimates........ 26
**Figure 18** Scatter plot of 53 SNPs associated with CHD, and educational outcomes ........ 27
**Figure 19** Scatter plot of 53 SNPs associated with CHD, and educational outcomes (with MR-Egger) ........ 28
**Figure 20** Funnel plot of 53 SNPs, showing CHD instrument strength against causal estimate on education ... 29

**Supplementary references**........ 30

## SUPPLEMENTARY METHODS

### 1. Traditional observational analyses

The association between education and self-reported CHD prevalence was derived from NHANES data using multivariable logistic regression adjusting for age and sex (**Supplementary Table 1**).(1) The association between education and the incidence of clinically-verified CHD was derived from two data sources. First, prospective data from the HAPIEE study was analysed with a Cox proportional hazard regression adjusting for age, sex, and country.(2) Second, we reanalysed published summary-level data from the MORGAM consortium, where the published hazard ratios (HR) were initially expressed in terms of country-specific top vs. bottom tertiles of education.(3) Here, we took the log of the hazard ratios corresponding to the lowest and highest educational tertiles, and divided it by the number of years of education between the lowest and highest tertiles from another publication,(4) and inverted the sign (to express the estimate per 1-year of additional education). Results were multiplied by 1-SD (3.6 years of education), and log HRs were back transformed into HR of CHD per 1-SD increase in education. Country and sex-specific MORGAM estimates were then pooled using fixed-effects inverse-variance meta-analysis. HAPIEE and MORGAM estimates were meta-analysed similarly, to derive a summary estimate of incidence.

As our MR analyses assumed a linear relationship between education and CHD, we used individual-level data from NHANES and HAPIEE to model the dose-response observational association between years of education (as an ordinal categorical variable) and risk of CHD (**Supplementary Figures 10-11**).

### 2. Genetic correlation between education and CHD

To investigate the genetic correlation between education and CHD, we used the "Lookup Center" function of the LD Hub platform (http://ldsc.broadinstitute.org).(5) On 28th October 2016, we downloaded an XLS file of genetic correlations, based on latest data that has been previously uploaded onto the LD Hub platform. Analyses were done by Linkage Disequilibrium Score Regression.

### 3. Causal analyses

#### 3.1. Conventional Mendelian randomization

To avoid biases due to overlapping datasets (i.e. where data from the gene-education association and gene-CHD association are derived from the same samples, and which can lead to bias in the direction of the observational association in the presence of a weak instrument),(6) we excluded data from the following studies from the SNP-education data source: deCODE, WTCCC, KORA, THISEAS, and 23andMe.(7) The SNP-education estimates from this restricted dataset (n= 349,306) were highly correlated with the SNP-education estimates from the complete SSGAC dataset (Pearson's r for 162 SNPs=0.96 [p-value<0.001] and Pearson's r for 72 SNPs=0.92 [p-value<0.001]) (**Supplementary Figures 5 & 6**), and used in subsequent MR analyses. **Supplementary Dataset 1** gives details for each SNP from both overlapping and nonoverlapping data sources.

**Supplementary figures 7 & 8** describe the matching procedure between SNPs retrieved from education GWAS and CARDIoGRAMplusC4D for the two sets of 162 and 72 SNPs, respectively. Where necessary, proxies were retrieved using the SNP Annotation and Proxy Search online tool (SNAP, http://archive.broadinstitute.org/mpg/snap/ldsearch.php; reference panel = 1000 Genomes; LD threshold r2>0.80).(8)

For all MR analyses, alleles from SSGAC and CARDIoGRAMplusC4D datasets were aligned to correspond to an increase in educational attainment. Conventional MR analysis was conducted using the inverse-variance weighted (IVW) approach, i.e. a linear regression of the SNPs-education estimates on SNPs-CHD estimates, weighted by the minor allele frequencies of each SNP and forced to pass through the origin.(9)

We estimated the power for the conventional MR analysis to detect the same magnitude of association reported in the observational studies, using a two-sided alpha of 0.05. Power was ≥98% for both sets of genetic instruments (**Supplementary Table 2**).

## 3.2 Sensitivity Mendelian randomization analyses

Sensitivity analyses investigated the potential presence of unbalanced horizontal pleiotropy among the genetic variants under analysis.

### *3.2.1. Penalized weighted median Mendelian randomization*

A penalized weighted median MR analysis was conducted (implemented in Stata using the *mrrobust* package; available at: https://github.com/remlapmot/mrrobust).(10) This gives more weight to genetic variants close to the median causal estimate. Weighted Median methods yield robust and precise results even when up to 50% of the weight in the analysis stems from invalid genetic variants.(10)

### *3.2.2. MR-Egger regression*

MR-Egger regression was applied as described by Bowden et al.(9) Based on the same principles as the Egger test (which assesses small study bias in meta-analysis) the method is similar to conventional MR analyses. However, the regression is not constrained to pass through the origin. A significant departure of the y-intercept from the origin gives evidence for the presence of unbalanced pleiotropy. If the level of pleiotropy is independent of the strength of the association between SNPs and the exposure under analysis, the MR-Egger estimate thus represents the true causal effect, even if all the genetic variants present pleiotropic effects (as per the InSIDE rule).(9) The standard error (SE) of the causal estimate was corrected by dividing the reported SE of the estimate by the residual SE.

*Additional sensitivity analysis (Supplement only)*

### *3.2.3. MR-Egger +SIMEX*

All MR approaches rely on the fact that the SNP-exposure association is true (NO Measurement Error [NOME] assumption), but whenever the SNP-exposure association estimates are spurious (violation of the NOME assumption), weak instrument bias can distort the causal effect estimate (specifically diluting it towards the null value).(11) Using the I2 statistic, we thus quantified the expected dilution in the MR-Egger causal effect estimates due to the variance of the estimates of the SNP-education association: I2 was only moderate for the set of 162 SNPs (I2=66%; potential dilution of 44%), whereas I2 for the set of 72 SNPs indicated a reduced risk of bias (I2=93%; potential dilution of 7%). As described by Bowden et al, we applied simulation extrapolation (SIMEX; implemented in R using the *simex* package) to adjust the MR-Egger causal estimates to account for violations to the NOME assumption (NO Measurement Error; results based on 10,000 simulations are presented in **Supplementary Table 4**).(11)

### *3.2.4. Mode Based Methods (assuming Zero Modal Pleiotropy)*

It is also possible to assess the potential role of horizontal unbalanced pleiotropy through recently developed methods that relax the conventional MR assumptions, and instead form a less stringent assumption of *Zero Modal Pleiotropy.* This postulates that pleiotropic SNPs are unlikely to converge on the same modal (most common) estimate due to pleiotropic effects not being identical. In contrast, valid SNPs are more likely to converge on the same, common modal estimate. We performed three analyses to exploit this assumption:

#### 3.2.4.1. Mode-Based Estimate

In our first analysis, we used the Mode-Based Estimate (MBE). With an infinite sample (i.e. no measurement error), the MBE would use the modal causal estimate (i.e. the most common instrumental variable estimate out of the 162 SNPs, where the instrumental variable estimate for each SNP is derived by dividing the SNP to CHD estimate by the SNP to education estimate). In finite samples, the MBE uses the mode of the smoothed empirical density function of causal estimates (where the instrumental variable estimate for each SNP is upweighted by its relative precision, in comparison to other SNPs). A tuning parameter $\varphi$ regulates the bias-variance trade-off. We explored a range of these, following which $\varphi$=0.5 was chosen to best fit the data. The analysis makes the assumption that the most commonly observed causal effect estimate comes from valid genetic instruments, and it can provide a consistent causal effect estimate even if the majority of (non-modal) genetic instruments are invalid.

One advantage of the Mode-Based Estimate is that it is less influenced by outlying (and possibly pleiotropic) genetic instruments without formally removing them from the analyses, thus making full use of the data. However, the

uncertainty around the point estimate can sometimes be prohibitively wide. For this reason, we exploited the *Zero Modal Pleiotropy* assumption using another strategy, which involves actually removing some genetic instruments from the analysis.

3.2.4.2. Largest Homogeneous Subset-MR

In our second analysis, SNPs were removed, one-by-one, until the final set of SNPs contained only sufficiently similar (according to some criteria) effect estimates. As such this final set of SNPs can be thought of as a relatively "homogeneous subset". The steps we took are:

1. Begin with the set of 162 SNPs. Evaluate the heterogeneity among each of the 162 causal estimates, using *Cochran's Q* statistic.(12)
2. Remove the SNP which contributes most to heterogeneity.
3. Repeat the proves, until a P-value threshold of heterogeneity is reached (e.g. P>0.05). Smaller P-values denote more heterogeneity (close to the original set of 162 SNPs) while larger P values include fewer SNPs and are hence more stringent.

3.2.4.3. Largest Homogeneous Subset-MR: removing *most causal* variants

Our third analysis was similar to the second one described above. This time, instead of removing any SNP (either at the left- or right-hand tail of the causal effect distribution) we only removed those SNPs that provided the strongest causal estimates (on one side of the tail), until the heterogeneity P value was attained as above. This method is unlikely to provide a valid causal estimate, as its result will be biased towards the null. However, it is an extreme example of a very stringent sensitivity check which asks the question *"What if the most outlying SNPs, all of which produce the strongest causal estimates, were deemed as invalid (due to having suspected horizontal, unbalanced pleiotropic effects on CHD)?"*

Results from all three Mode Based Methods are presented in **Supplementary Table 4**. To summarize these findings, the first Mode-Based Estimate yielded directionally concordant point estimates. However, this test was grossly underpowered to detect a causal effect. The Largest Heterogeneous Subset analyses, by contrast, were better powered. The vast majority of SNPs (i.e. 90%) were highly homogeneous in their causal effect estimates. Removing these 0-10% heterogeneous SNPs made little difference to the point estimates, and furthermore all overall MR estimates retained conventional levels of statistical significance, so were unlikely to have been observed by chance alone. Altogether, findings from the three mode based analyses were consistent with those from the main IVW analyses, and with the hypothesis of limited confounding from unbalanced horizontal pleiotropy.

***3.2.4. Causal association between genetic liabilities for CHD and education***

Since genetic liabilities for CHD may also influence educational attainment already at earlier ages, we tested whether the genetic risk of CHD was associated with educational attainment. We used data from the CARDIoGRAMplusC4D Consortium to extract SNP-CHD estimates for 53 independent SNPs (at r2<0.02) that were GWAS significant (P<5x10-8).(13-16) We directly matched these with the corresponding SNPs from the SSGAC GWAS, involving 328,917 individuals (**Supplementary Dataset 3**).(7) The analyses were conducted similar to the analyses described above. This analysis was performed using data where the underlying participants overlapped slightly between the SNP-exposure and SNP-outcome estimates. However, as such overlap biases results away from the null,(17) and since our finding was quite definitively null, we did not purse seeking non-overlapping data in this instance, for this particular sensitivity analysis

## 3.3. Causal relationships from education to 10 cardiovascular risk factors, from 6 GWAS consortia

The conventional MR approach was used to identify associations between genetic predisposition towards higher educational attainment and cardiovascular risk factors that could be potential mediators. Outcome data were taken from various publicly available datasets: ever smokers vs. never smokers (from the Tobacco and Genetics Consortium);(18) blood pressure (International Consortium for Blood Pressure);(19) LDL-cholesterol, HDL-cholesterol and triglycerides (Global Lipids Genetic Consortium);(20) type 2 diabetes (DIAbetes Genetics Replication And Metaanalysis, stage 1 GWAS, version 3/2012Dec17);(21) glucose (Meta-Analyses of Glucose and Insulin-related traits Consortium);(22) body mass index (BMI) and height (Genetic Investigation of ANthropometric Traits).(23, 24)

Statistical analyses were conducted using Stata v.13. Simulation extrapolation analyses were conducted using R v3.3.1.

**Supplementary Table 1** Description of observational studies

| Study | The National Health and Nutrition Examination Survey (NHANES)(1) | Health, Alcohol and Psychosocial factors In Eastern Europe (HAPIEE)(2) | MOnica Risk, Genetics, Archiving and Monograph (MORGAM)(3) |
|---|---|---|---|
| **Access policy** | Public domain, with all required data instantly downloadable | Access upon application to principal investigators.<br>Our analysis has not been previously published. | Access upon application to principal investigators.<br>Our analysis is based on previously published work. |
| **Design** | Cross-sectional | Longitudinal | Longitudinal |
| **Country** | United States of America | Russia, Czech Republic, Poland, Lithuania | 9 European countries (Sweden, Finland, Denmark, Northern Ireland, Scotland, France, Germany, Italy, Lithuania) |
| **Baseline** | 1999-2014 (8 waves) | 2002-2008 | 1983-2004 |
| **Age at baseline** | 20-85 | 43-74 | 35-64 |
| **Initial sample** | 43,611 | 34,876 | unknown |
| **Exclusion criteria** | Not applicable | Self-reported hospitalization/diagnosis with AMI, stroke, coronary heart disease or angina. Or positive score on the Rose Angina Questionnaire | Documented or self-reported history of myocardial infarction or unstable angina pectoris |
| **Analytic sample** | 43,611 | 23,511 | 97,048 |
| **Incident/Prevalent CHD cases** | Prevalent | Incident | Incident |
| **Case ascertainment** | Self-reported history (hence non-fatal only) as per *"Has a doctor ever said you had a heart attack?"* or *"…coronary artery disease?".*<br>Sensitivity analyses further restricted to *"heart attack"* only (See Supplementary Figure 2) | National MI and mortality registries | National MI and mortality registries |
| **Cases (*n*)** | 1,933 | 309 (fatal) + 323 (non-fatal) = 623 total | 6 522 |
| **Follow-up (median)** | - | 6.9 years | 10.0 years |
| **Statistical model** | Logistic Regression | Cox Proportional Hazards Regression | Cox Proportional Hazards Regression within each country & gender, followed by meta-analysis (detailed in the "methods" section). |
| **Weighting/adjustment** | Adjusted for age, sex.<br>Weighted to account for non-random sampling, response bias and geographical clustering | Adjusted for age, sex, country | Adjusted for age |

SD, standard deviation. CHD, coronary heart disease. MI, myocardial infarction.

**Supplementary Table 2** Power for conventional Mendelian randomization analysis (two-sided α=0·05)

| Exposure/genetic instrument | R-squared (of variance in educational phenotype) | Actual N (CARDIoGRAMplusC4D) | Proportion of cases (CARDIoGRAMplusC4D) | Observational OR | N required for 80% power | Power at actual N |
|---|---|---|---|---|---|---|
| **Education/1st set of SNPs (162 SNPs)** | 0.018 | 194,427 | 0.327 | 0.8* | 42,832 | >0.99 |
| **Education/2nd set of SNPs (72 SNPs)** | 0.008 | 194,427 | 0.327 | 0.8* | 96,372 | 0.98 |

* based on traditional observational estimate of education and risk of incident CHD from meta-analysis of HAPIEE and MORGAM studies (see **Figure 2**)
Power calculation was based on the method developed by Brion et al.(25)

**Supplementary Table 3** Sensitivity analyses for observational estimates

| Study | Case definition/ sub-analysis | | Cases (*n*) | Controls (*n*) | Mean age at first event | Odds ratio (OR) / hazard ratio (HR) of CHD, per 1-SD higher education | Result taken forward into main results (presented in figure 1)? |
|---|---|---|---|---|---|---|---|
| ***PREVALENCE*** | | | | | | | |
| **NHANES*** | | | | | | | |
| | Nonfatal CHD | | | | | | |
| | | All ages | 2,846 | 40,823 | 55.1 | OR = 0.73 (0.68; 0.78) | Yes |
| | | All ages (no missing data, to compare with SES-adjusted estimate below) | 1,234 | 16,790 | 55.1 | OR = 0.75 (0.67; 0.83) | |
| | | All ages, fully SES-adjusted† | 1,234 | 16,790 | 55.1 | OR = 0.73 (0.62; 0.85) | |
| | | Age of first event <66y | 1,907 | 40,823 | 48.7 | OR = 0.72 (0.66; 0.78) | |
| | Nonfatal AMI only | | | | | | |
| | | All ages | 1,933 | 41,678 | 54.7 | OR = 0.71 (0.65; 0.77) | |
| ***INCIDENCE*** | | | | | | | |
| **HAPIEE‡** | | | | | | | |
| | Fatal / nonfatal CHD event | | 632 | 22,879 | 65.0 | HR = 0.75 (0.69; 0.81) | Yes (meta-analysed) |
| | Nonfatal CHD event only | | 338 | 23,138 | 64.1 | HR = 0.81 (0.72; 0.91) | |
| | Fatal CHD event only | | 309 | 23,202 | 67.2 | HR = 0.71 (0.64; 0.79) | |
| | Fatal CVD event | | 621 | 22,890 | 67.4 | HR = 0.75 (0.70; 0.82) | |
| **MORGAM‡** | | | | | | | |
| | Fatal / nonfatal CHD event | | 6 522 | 90 526 | 63.2 | HR = 0.83 (0.80; 0.86) | Yes (meta-analysed) |

* Adjusted for age and sex (additionally weighted to account for oversampled design, response rate, and geographical clustering).
† Adjusted for age, sex, ethnicity, citizenship, country of birth, military service, marital status, household size, family income: poverty threshold ratio.
‡ Adjusted for age, sex, and country of survey.
SD, standard deviation. CHD, coronary heart disease. AMI, acute myocardial infarction.

**Supplementary Table 4** Sensitivity analyses of Mendelian Randomization estimates.

| **Analysis** | **Causal effect (OR) estimate for risk of CHD (95% CI)** | **Causal effect P-value** | **MR-Egger constant (Log OR) (95% CI)** | **Egger test for pleiotropy (P-value)** |
|---|---|---|---|---|
| **Set of 162 SNPs** ($I^2$ statistic=0.661) | | | | |
| Conventional MR (IVW) | 0.67 (0.59 to 0.77) | $2.9 \times 10^{-8}$ | - | - |
| Weighted-Median MR | 0.70 (0.58 to 0.85) | $1.8 \times 10^{-4}$ | - | - |
| Standard MR-Egger | 0.54 (0.31 to 0.93) | 0.029 | 0.004 (-0.056 to 0.013) | 0.417 |
| Adjusted MR-Egger (+SIMEX) | 0.41 (0.19 to 0.87) | 0.022 | - | - |
| Mode-Based Estimate | 0.84 (0.44 to 1.60) | 0.255 | | |
| Largest Homogeneous Subset-MR (3 tests below): | | | | |
| Minus 2 most heterogeneous SNPs = 160 SNPs (Heterogeneity P≥0.05) | 0.68 (0.59 to 0.77) | $1.8 \times 10^{-8}$ | - | - |
| Minus 4 most heterogeneous SNPs = 158 SNPs (Heterogeneity P≥0.20) | 0.66 (0.59 to 0.75) | $8.8 \times 10^{-10}$ | - | - |
| Minus 12 most heterogeneous *causal* SNPs = 150 SNPs (Heterogeneity P≥0.20) | 0.75 (0.67 to 0.86) | $1.8 \times 10^{-5}$ | | |
| Minus (47 proxies) = 115 SNPs. Conventional MR estimate (IVW): | 0.62 (0.52 to 0.73) | $1.3 \times 10^{-7}$ | - | - |
| Minus (21 with >10% missing data) = 141 SNPs. Conventional MR estimate (IVW): | 0.70 (0.60 to 0.80) | $1.4 \times 10^{-6}$ | - | - |
| | | | | |
| **Set of 72 SNPs** ($I^2$ statistic=0.934) | | | | |
| Conventional MR (IVW) | 0.60 (0.49 to 0.74) | $6.2 \times 10^{-6}$ | - | - |
| Weighted-Median MR | 0.71 (0.54 to 0.93) | 0.014 | | |
| Standard MR-Egger | 0.54 (0.26 to 1.11) | 0.099 | 0.002 (-0.010 to 0.014) | 0.764 |
| Adjusted MR-Egger (+SIMEX) | 0.43 (0.17 to 1.12) | 0.088 | - | - |
| Mode-Based Estimate | 0.78 (0.40 to 1.54) | 0.490 | | |
| Largest Homogeneous Subset-MR (3 tests below): | | | | |
| Minus 2 most heterogeneous SNPs = 70 SNPs (Heterogeneity P≥0.05) | 0.65 (0.53 to 0.79) | $5.8 \times 10^{-5}$ | | |
| Minus 4 most heterogeneous SNPs = 68 SNPs (Heterogeneity P≥0.20) | 0.64 (0.53 to 0.78) | $9.4 \times 10^{-5}$ | | |
| Minus 5 most heterogeneous *causal* SNPs = 67 SNPs (Heterogeneity P≥0.20) | 0.67 (0.55 to 0.81) | $7.6 \times 10^{-5}$ | - | - |

All causal effects are expressed as change in Odds Ratio of coronary heart disease (CHD), per 3.6 years (1-SD) of longer education.
The adjusted MR-Egger regression estimates are the results of 10,000 simulations.
Section 3.2 (on page 4) of this Supplement file provides further methodological details.

SNP, single nucleotide polymorphism. MR, Mendelian randomization. IVW, inverse-variance weighted (analysis). SIMEX, simulation extrapolation.

**Supplementary Figure 1** Theoretical illustration of pleiotropic phenomena on estimates derived from Mendelian randomization (MR) analyses

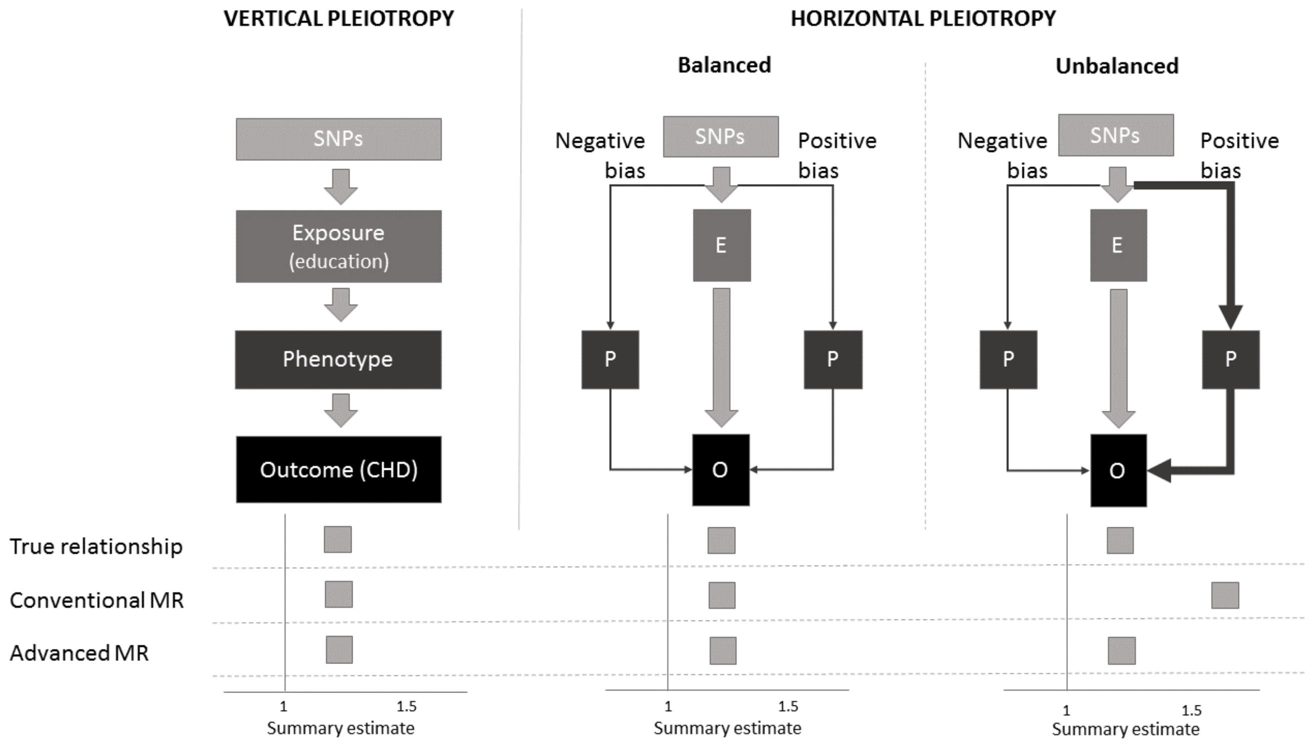


In the case of vertical pleiotropy, the conventional MR assumptions are satisfied as the intermediating phenotype lies on a single causal pathway. In the case of horizontal pleiotropy, one or more phenotypes lie on a different causal pathway. When the effects of the SNPs on the outcome through various intermediating phenotypes (including those on different causal pathways) are balanced, then estimates derived with conventional MR estimates should be valid. On the contrary, when the effects of SNPs on the outcome are systematically distorted towards one intermediating pathway (unbalanced horizontal pleiotropy), conventional MR estimates are invalid and biased. Advanced MR techniques (representing techniques such as MR-Egger and weighted median MR), accounting for presence of unbalanced pleiotropy of the genetic instrument, should nonetheless produce an estimate that is closer to the true underlying association between the risk factor and outcome.

Figure adapted from White J. *et al. JAMA Cardiol.* 2016.(26)

**Supplementary Figure 2** – Overview of the main steps in this study, showing existing datasets (grey), hypothesis formulation (blue), key findings (green), their interpretation (yellow), conclusion (orange) and final suggestions for discussion (red).

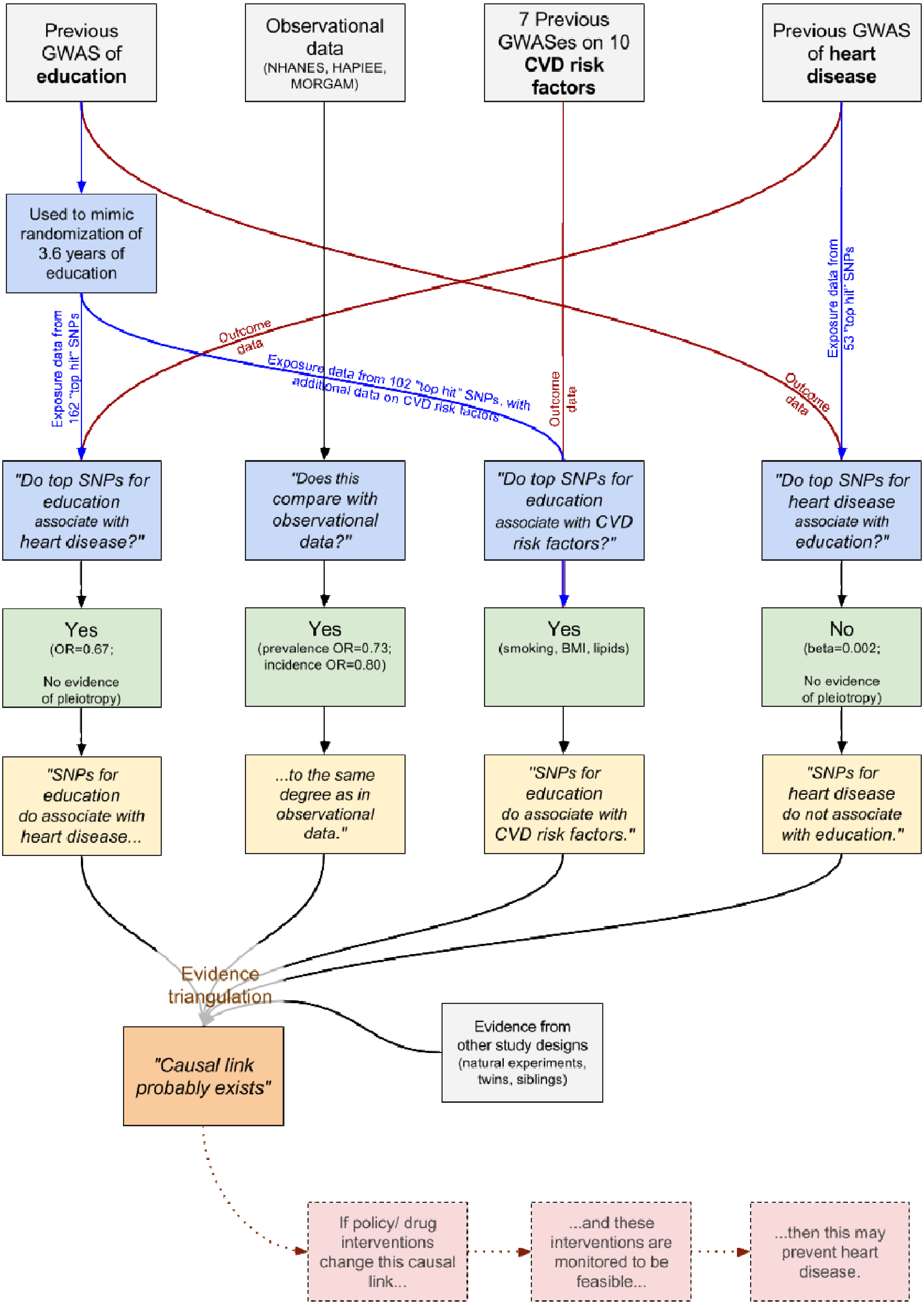

***Supplementary Figures 3-8, concerning METHODS:***

**Supplementary Figure 3** Flowchart of participants in observational NHANES analysis

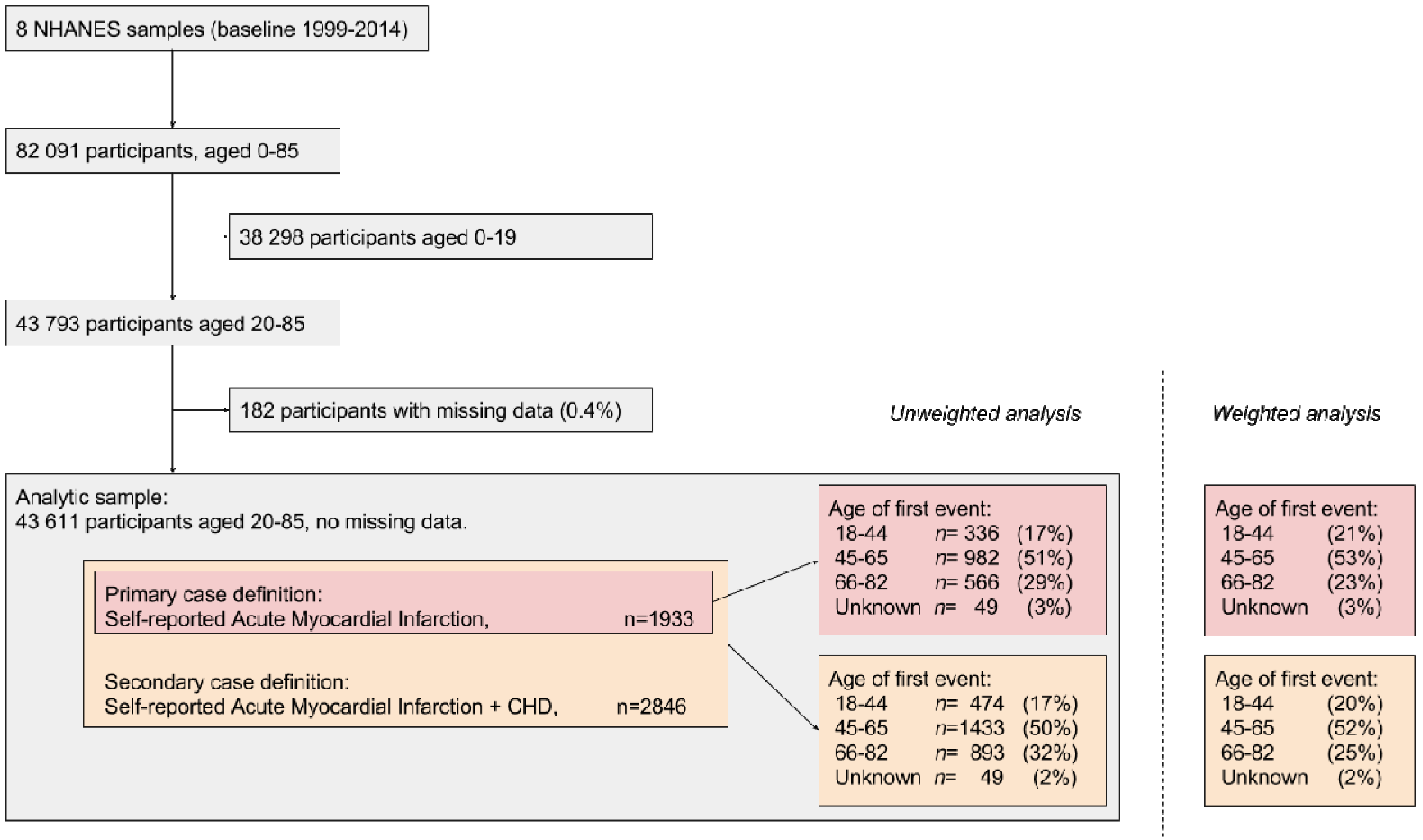

**Supplementary Figure 4** Flowchart explaining the derivation of the two sets of education SNPs (162 and 72 SNPs), used to estimate causal effects from education.

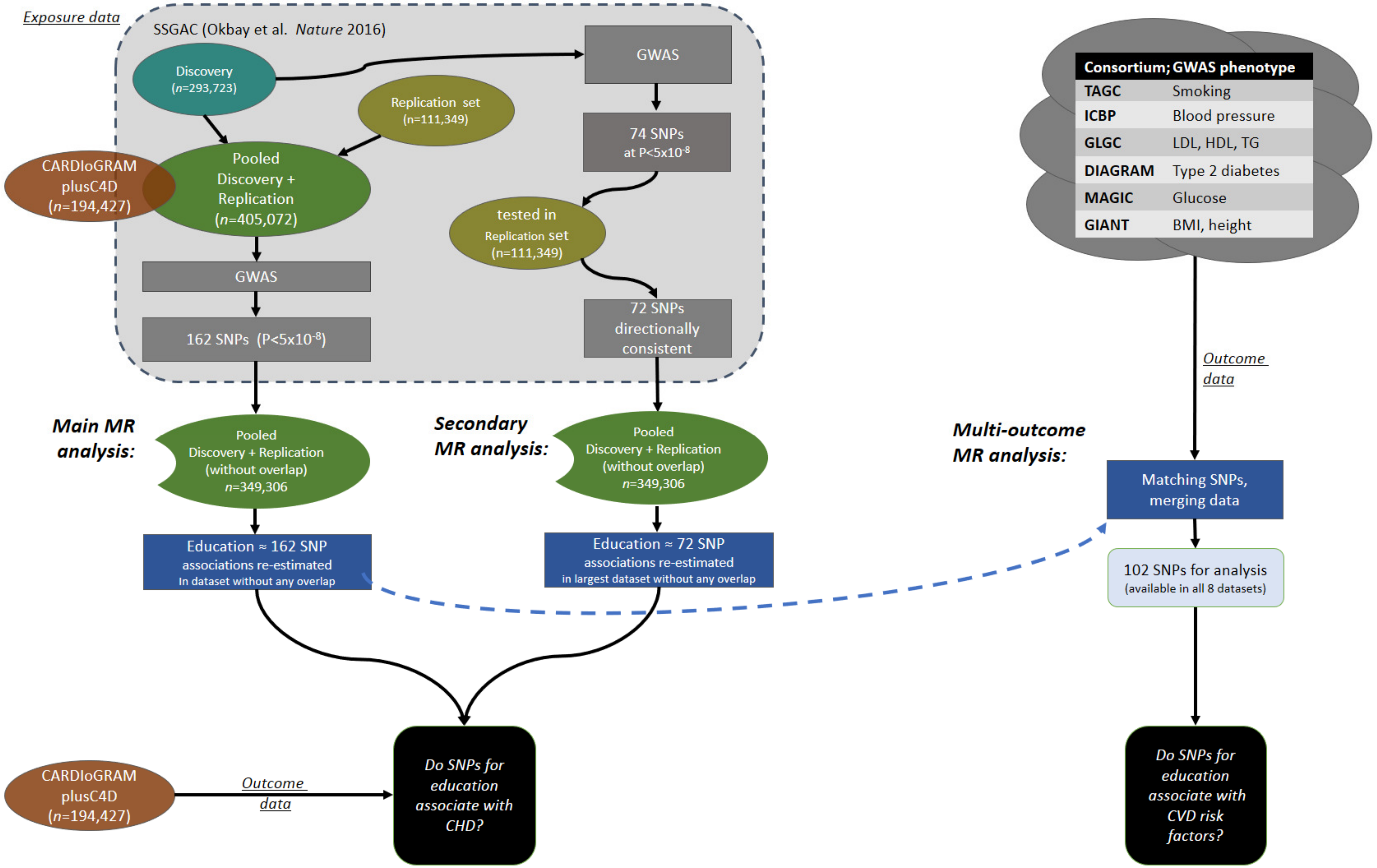

**Supplementary Figure 5** Scatter plot of SNP-education association estimates, comparing complete dataset vs. non-overlapping dataset (n=162 SNPs)

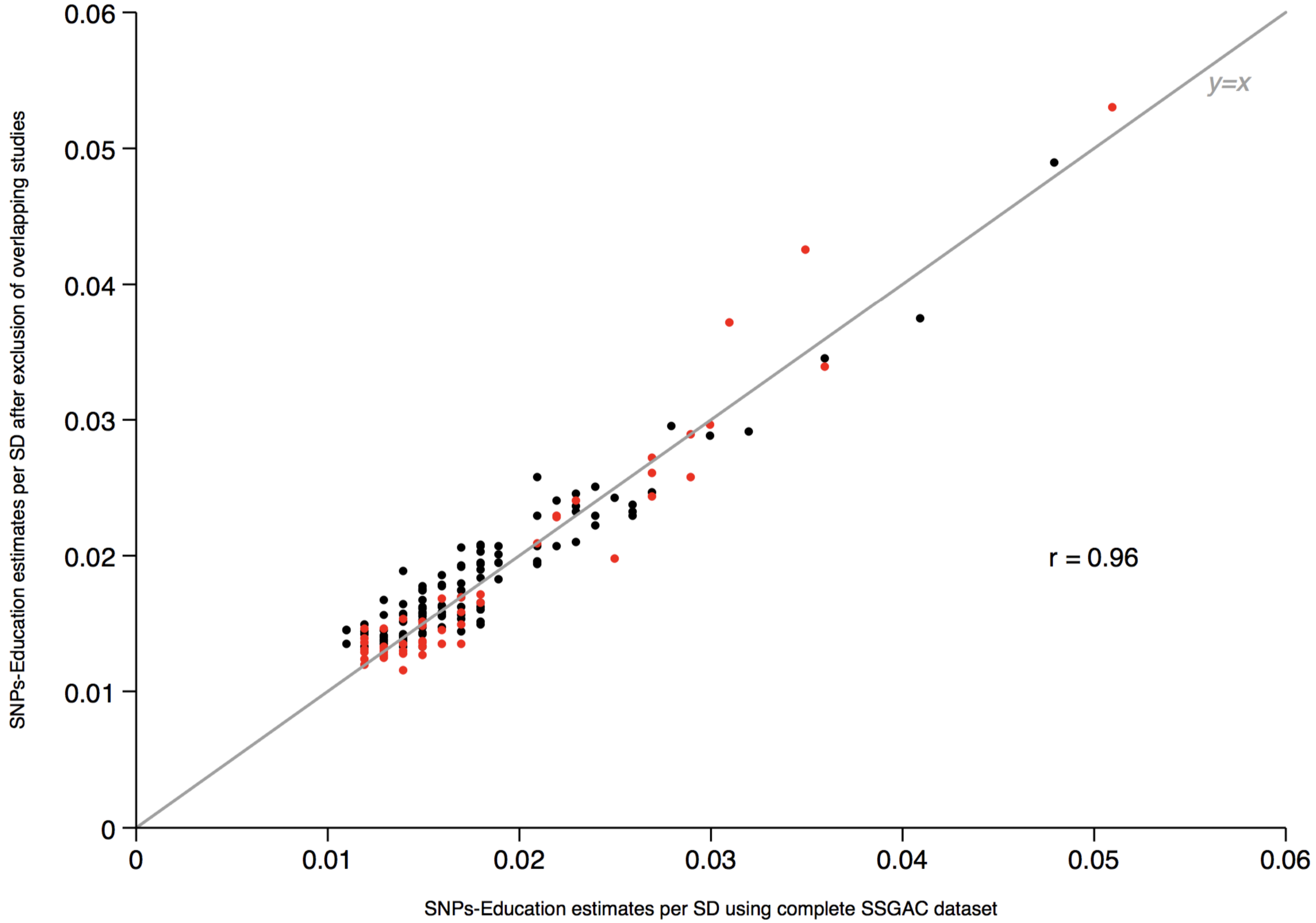


The complete SSGAC dataset (x-axis) was based on 405’072 participants. The restricted dataset (y-axis) was based on 349,306 participants without sample overlap. Median standard errors (SE) in the complete SSGAC and restricted dataset were both of 0.003. Red points indicate 51 SNPs that remained associated with the education phenotype above the GWA threshold (P>5 x $10^{-8}$) in the smaller dataset without sample overlap.

**Supplementary Figure 6** Scatter plot of SNP-education association estimates, comparing complete dataset vs. non-overlapping dataset (n=72 SNPs)

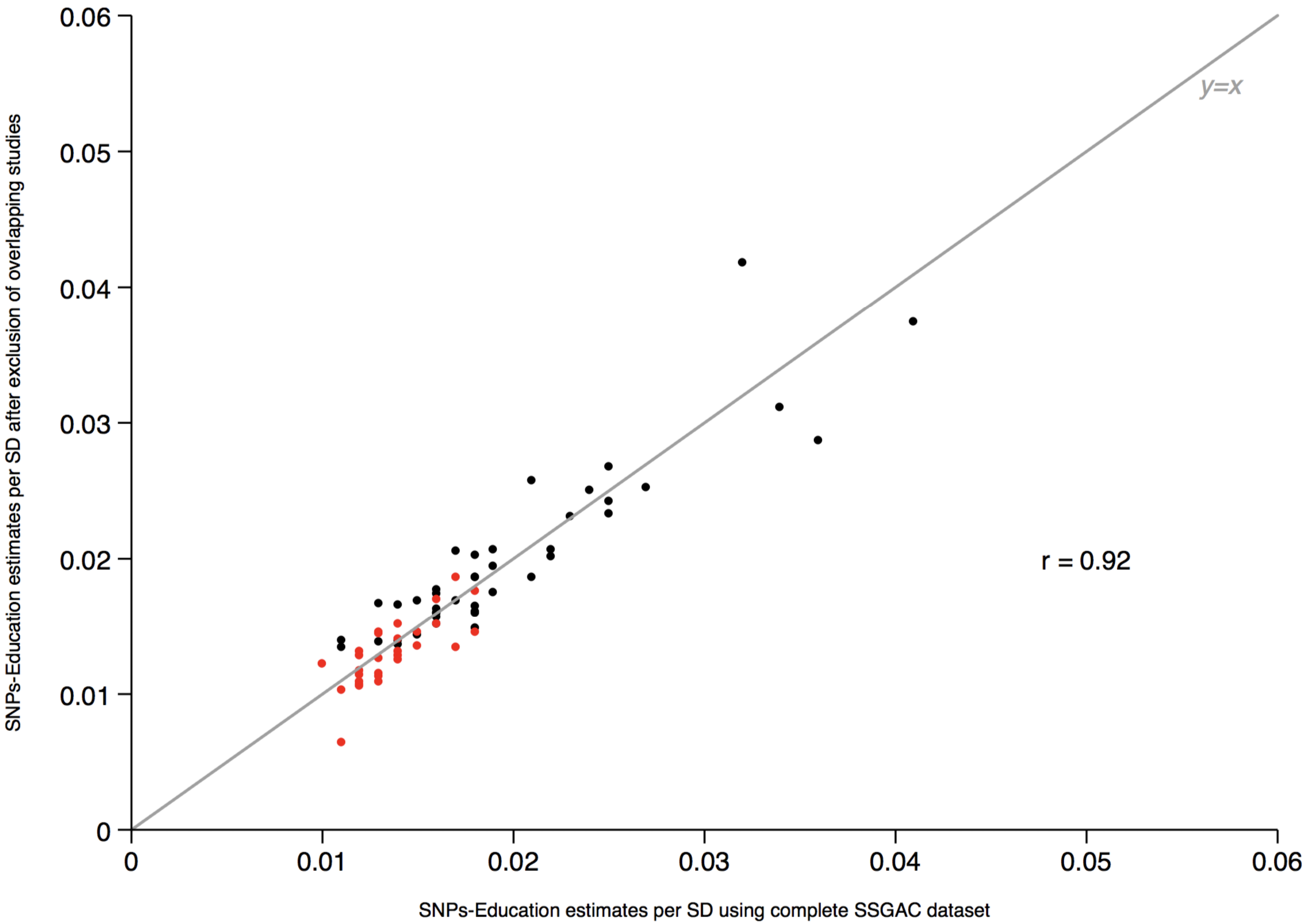


The complete SSGAC dataset (x-axis) was based on 293’723 participants. The restricted dataset (y-axis) was based on 349,306 participants without sample overlap (and where the replication data was additionally used, to allow for most precise estimates while concurrently minimizing bias). Median SE in the complete SSGAC and restricted data dataset were both of 0.003. Red points indicate 29 SNPs that remained associated with the education phenotype above the GWA threshold ($P>5 \times 10^{-8}$) in the dataset without sample overlap.

**Supplementary Figure 7** Flowchart illustrating how SNPs identified in the SSGAC education GWAS were mapped against SNPs reported in CARDIoGRAMplusC4D (n=162 SNPs)

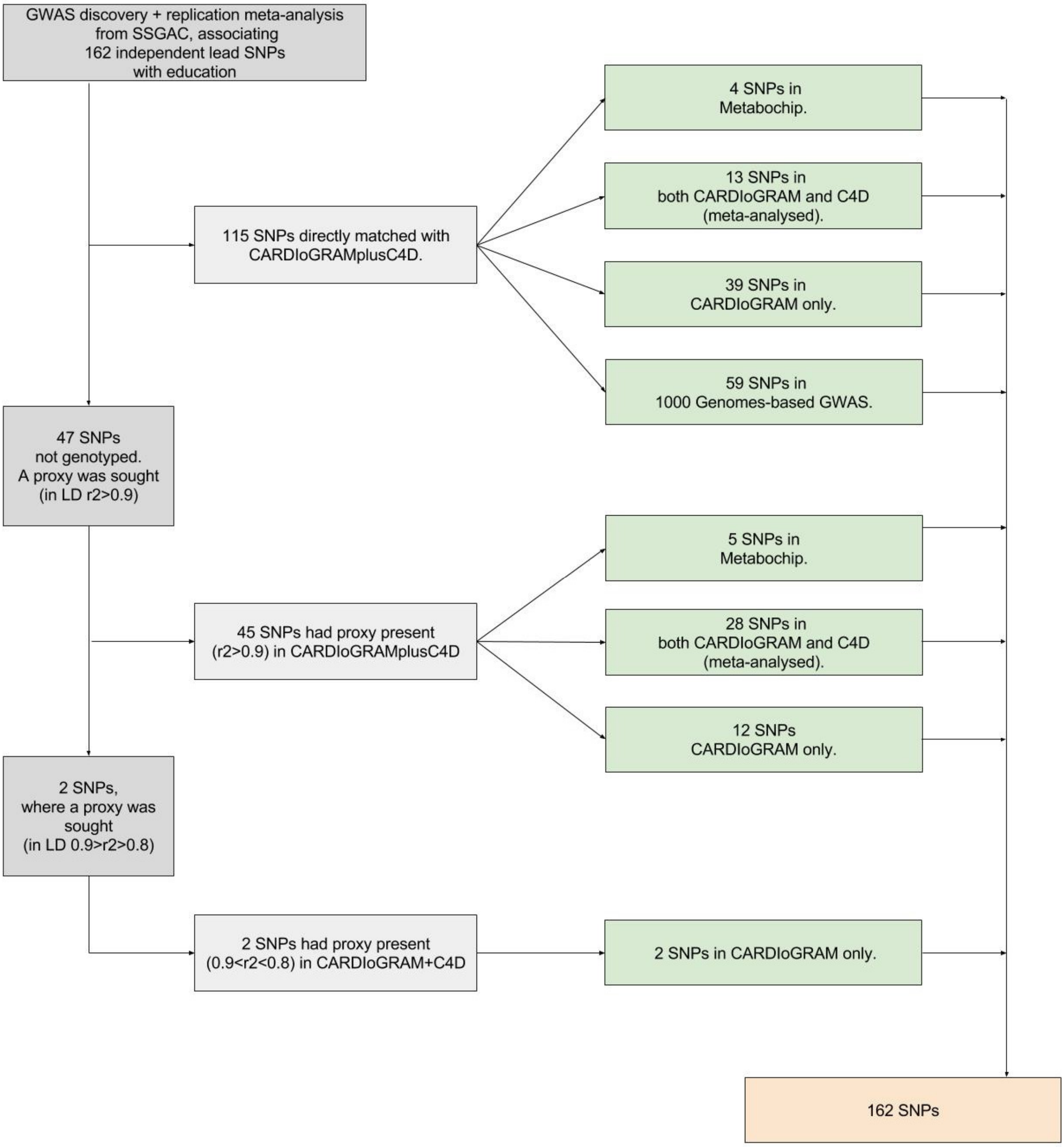


Where necessary, proxies were retrieved using the SNP Annotation and Proxy Search online tool (SNAP, http://archive.broadinstitute.org/mpg/snap/ldsearch.php ; reference panel = 1000 Genomes; LD threshold r2>0.80).

**Supplementary Figure 8** Flowchart illustrating how SNPs identified by SSGAC education GWAS were mapped against SNPs listed in CARDIoGRAMplusC4D (n=72 SNPs)

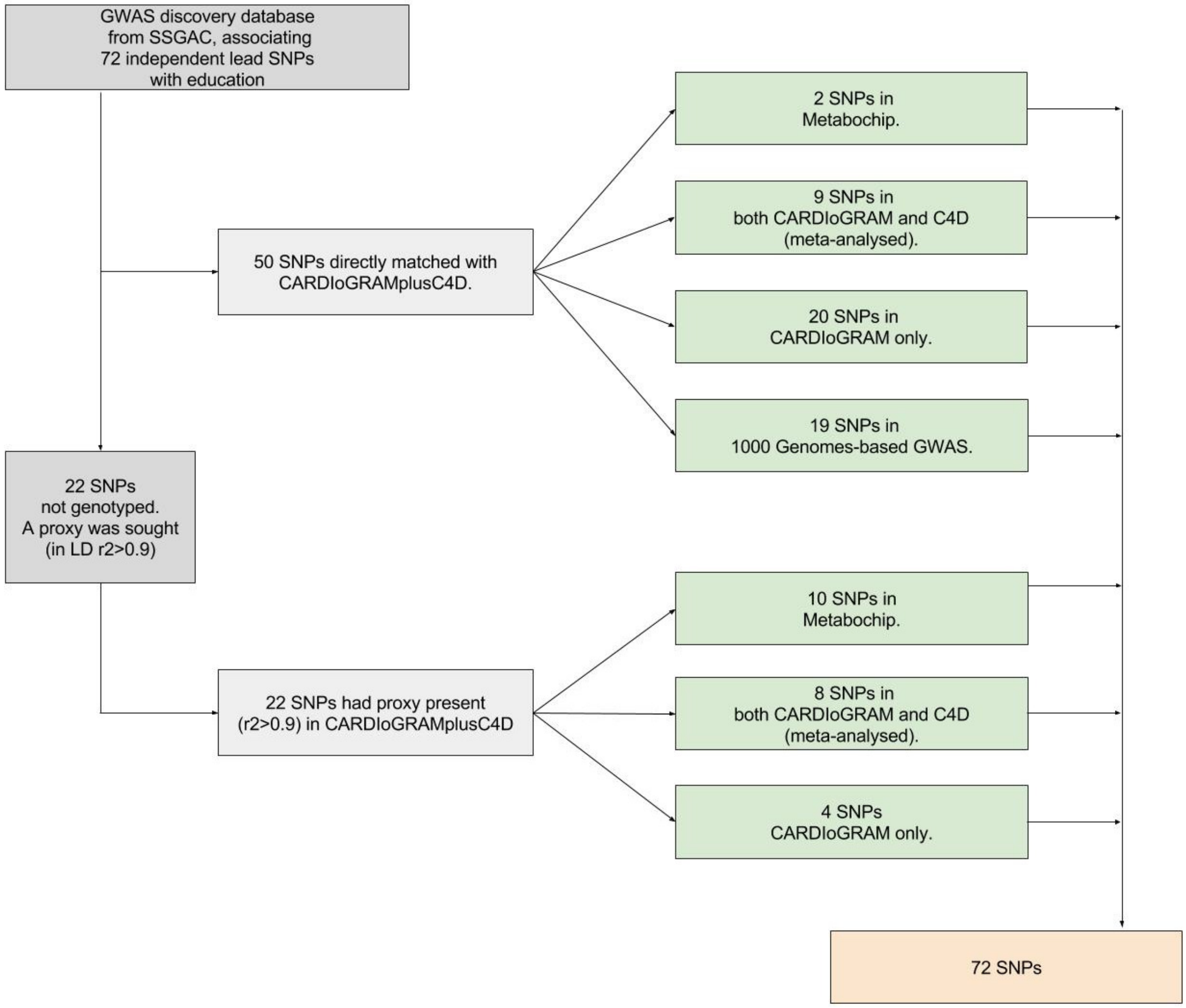


Where necessary, proxies were retrieved using the SNP Annotation and Proxy Search online tool (SNAP, http://archive.broadinstitute.org/mpg/snap/ldsearch.php ; reference panel = 1000 Genomes; LD threshold r2>0.80).

**Supplementary Figures 9-11, concerning OBSERVATIONAL RESULTS:**

**Supplementary Figure 9** Observational estimates from the MORGAM consortium, showing cohort-level estimates and the results from meta-analysis.

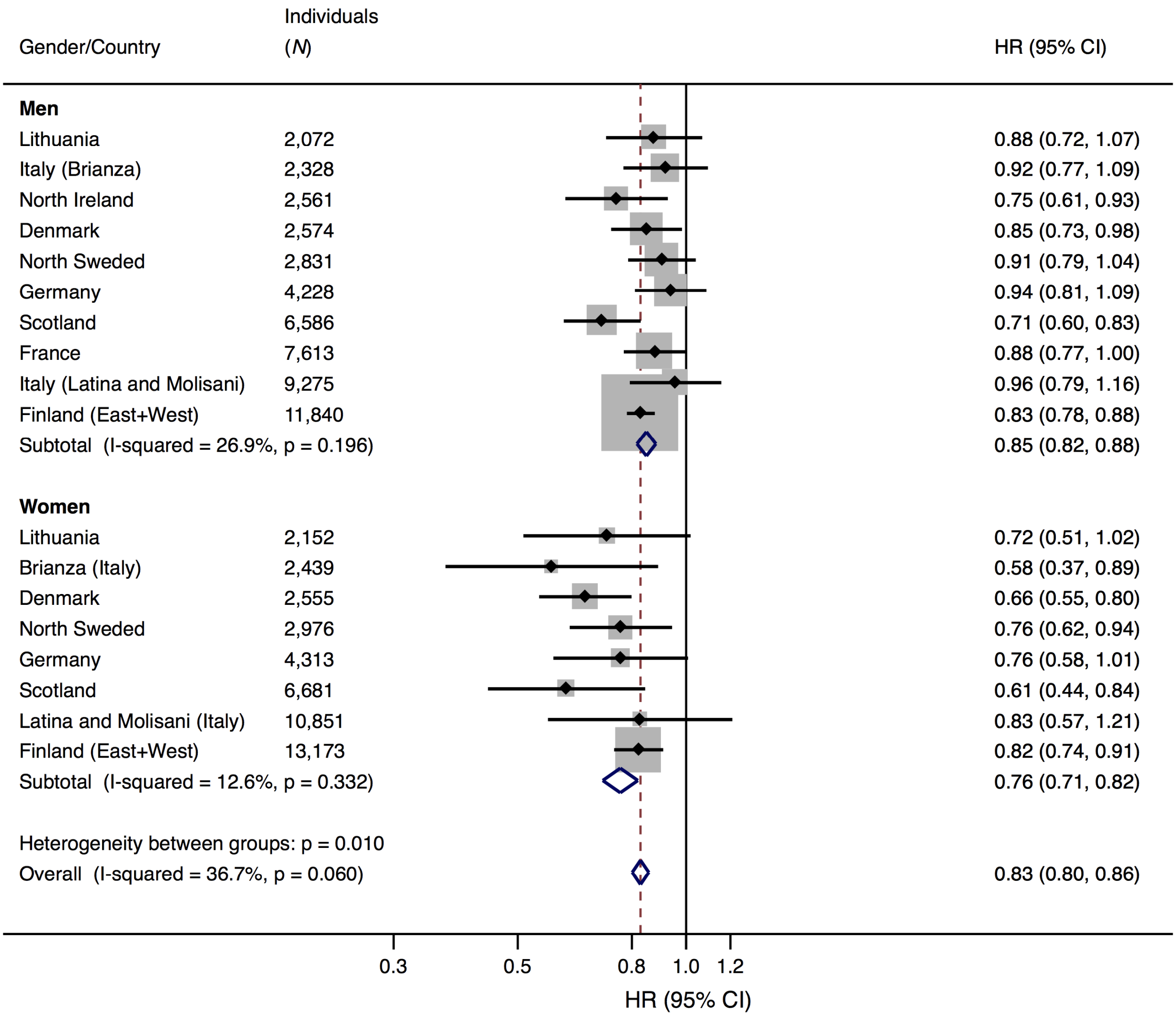


Meta-analysis performed using inverse-variance weighted fixed-effect modelling.
CHD, coronary heart disease. SD, standard deviation. HR, hazard ratio. CI, confidence interval.

**Supplementary Figure 10** Dose response relationship between education and CHD, using observational NHANES data

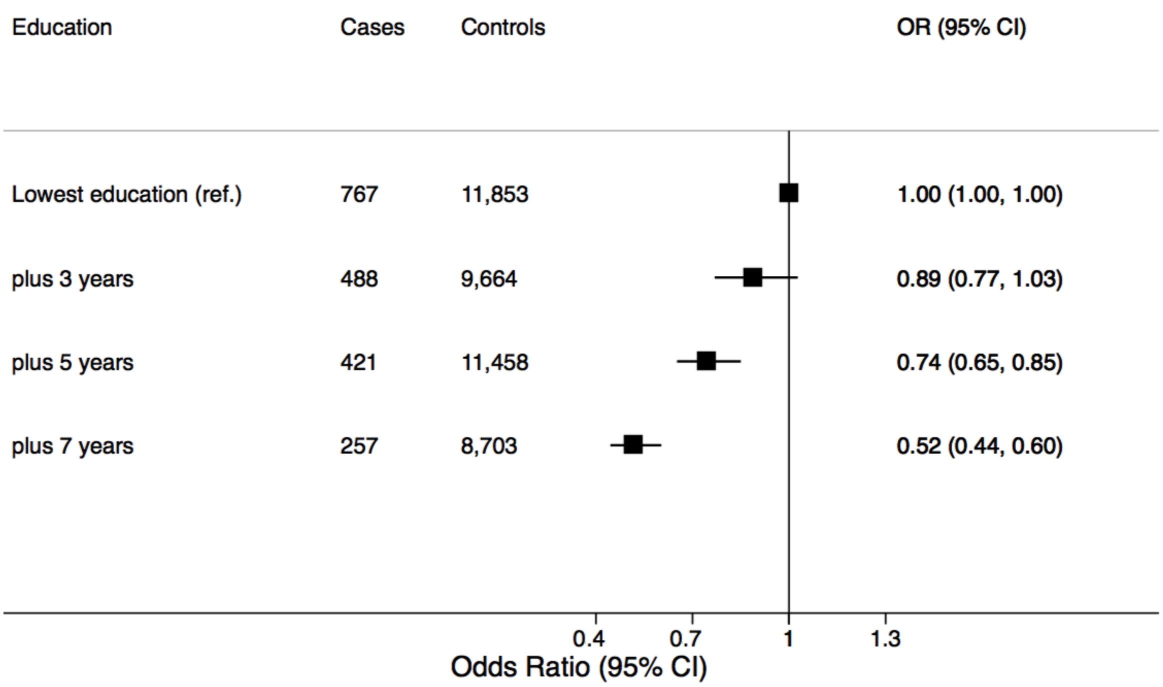


Lowest education group represents “some high school” in USA system, i.e. typically 16-year old pupil.
Logistic regression model was adjusted for age and sex.
P for trend < 0.0001.
CHD, coronary heart disease; NHANES, National Health and Nutrition Examination Survey;
OR, odds ratio CI, confidence interval.

**Supplementary Figure 11** Dose response relationship between education and CHD incidence, using observational HAPIEE data

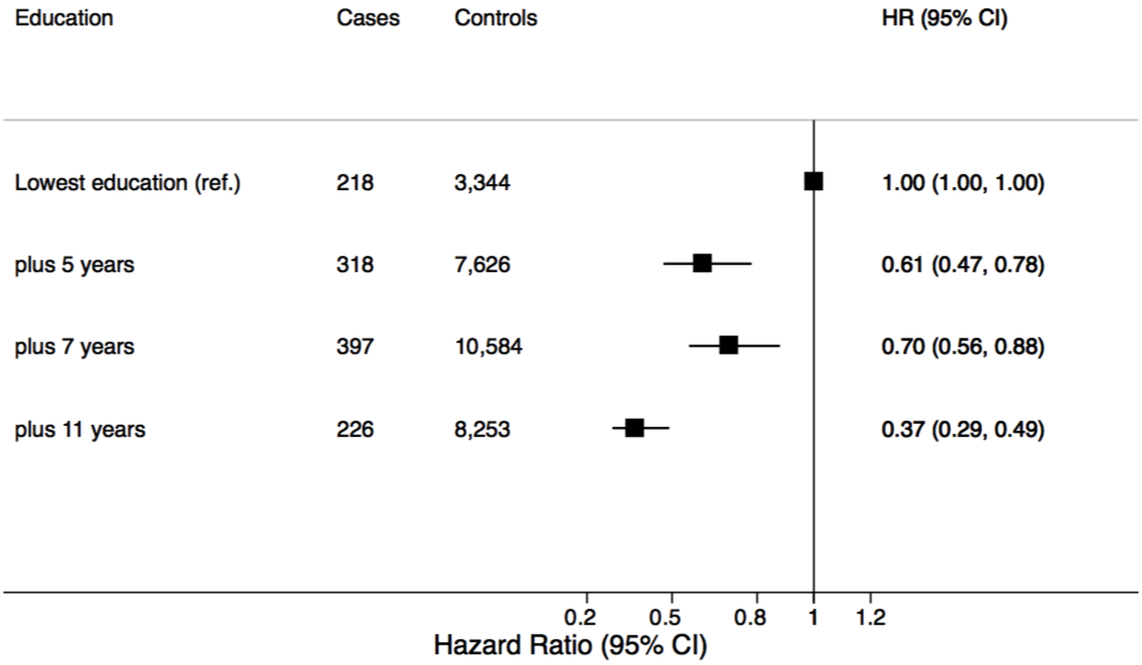


Lowest education group represents “Primary education or lower”, i.e. max. 4 years of education.
Cox proportional hazard regression model was adjusted for age, sex and country.
P for trend<0.001.
CHD, coronary heart disease; HAPIEE, The Health, Alcohol and Psychosocial factors In Eastern Europe Study; HR, hazard ratio CI, confidence interval.

**Supplementary Figures 12-, concerning CAUSAL RESULTS:**

**Supplementary Figure 12** Scatter plot of 162 SNPs associated with education and their risk of CHD (with 95% confidence intervals)

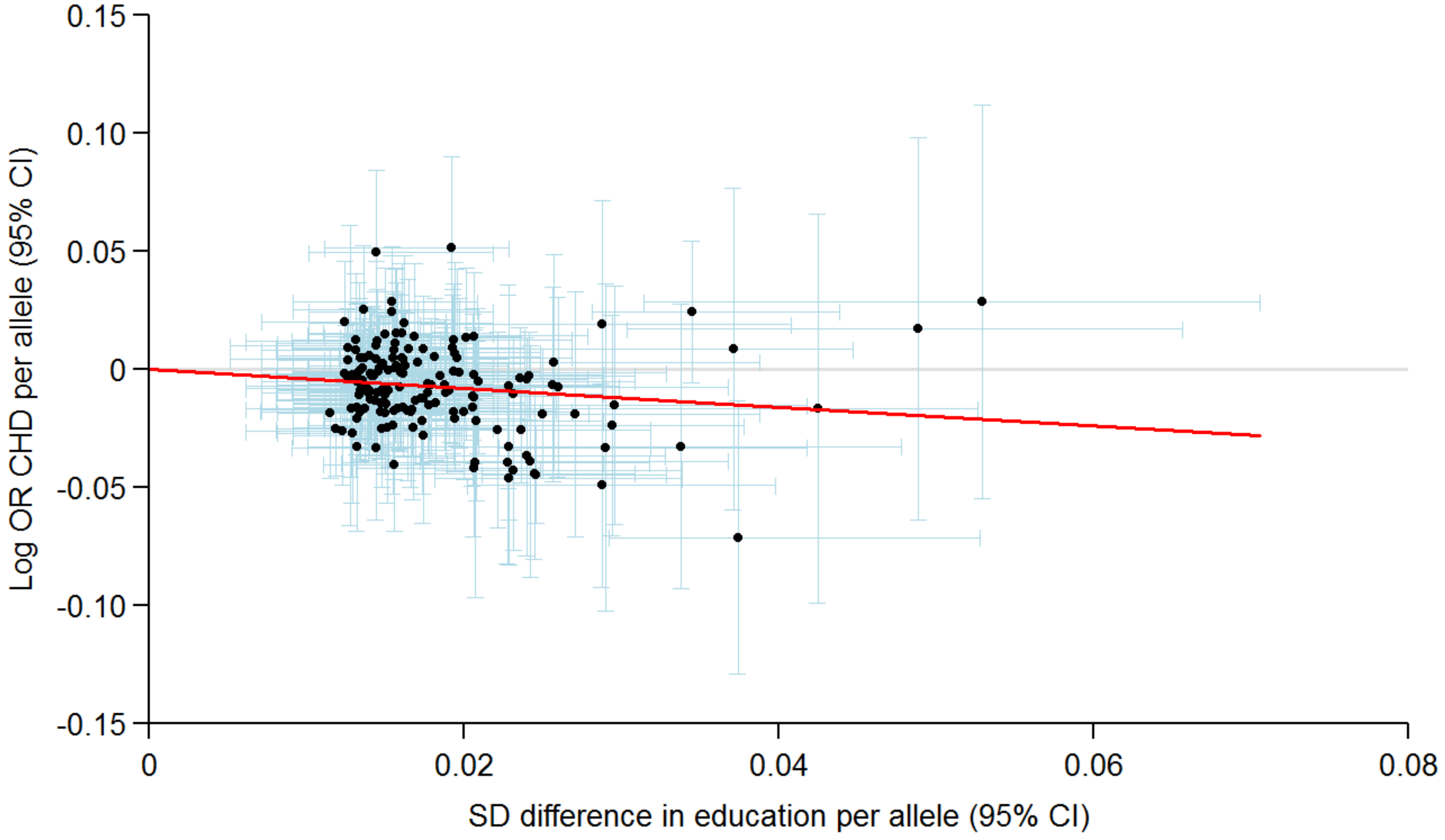


Each dot represents one single nucleotide polymorphism (SNP).
The red line represents the regression slope of the causal effect estimate of education on risk of CHD (where each SNP is weighted by its inverse allele frequency).
CHD, coronary heart disease. OR, odds ratio. CI, confidence interval.

**Supplementary Figure 13** Scatter plot of 162 SNPs associated with education and their risk of CHD (with MR-Egger)

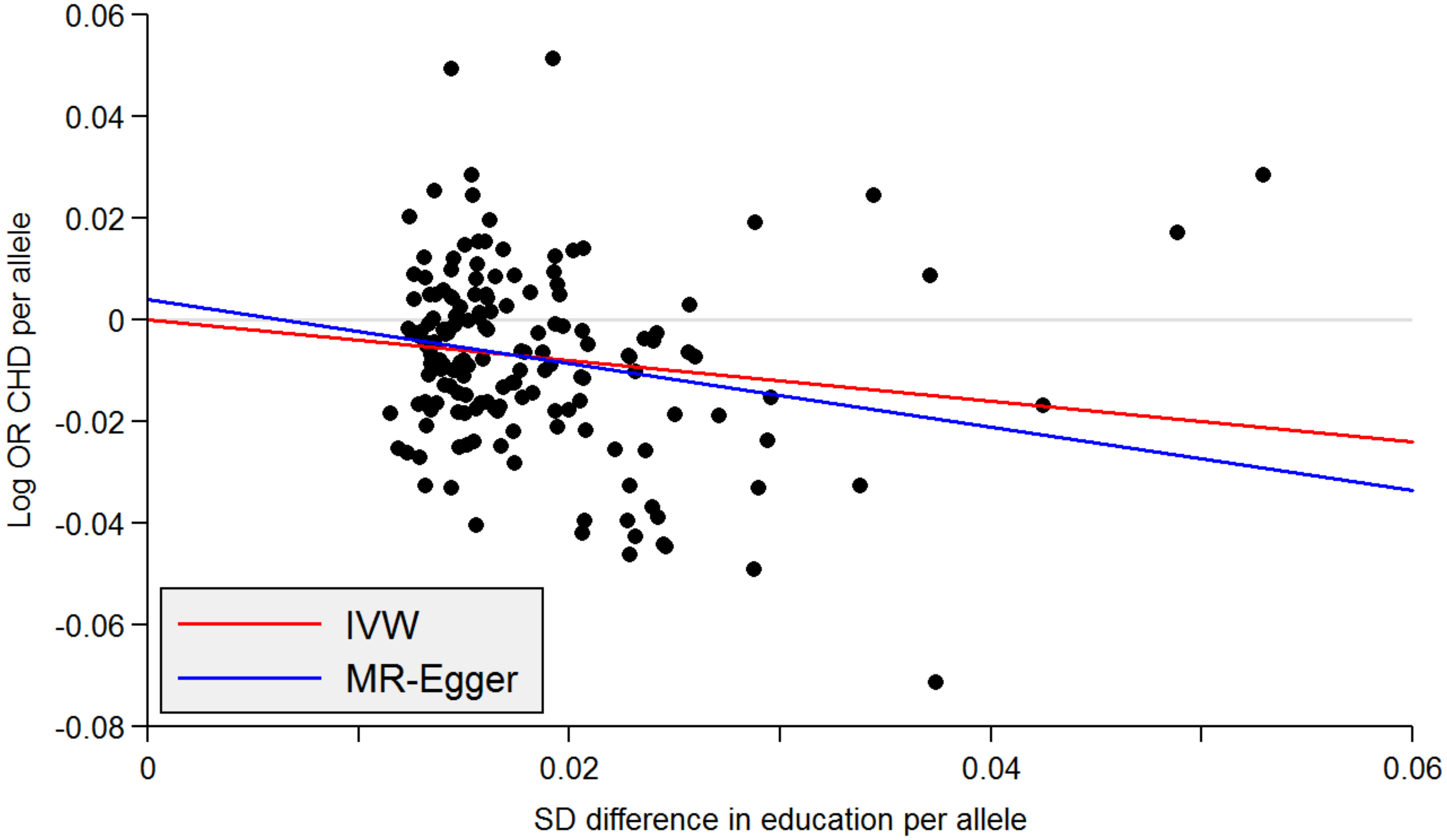


The red line shows causal regression estimates from conventional Mendelian randomization (MR), inverse variance weighted (IVW).
The blue line shows causal regression estimates from MR-Egger.
SNP, single nucleotide polymorphism. CHD, coronary heart disease. OR, odds ratio.

**Supplementary Figure 14** Funnel plot of 162 SNPS, showing instrument strength against causal estimates

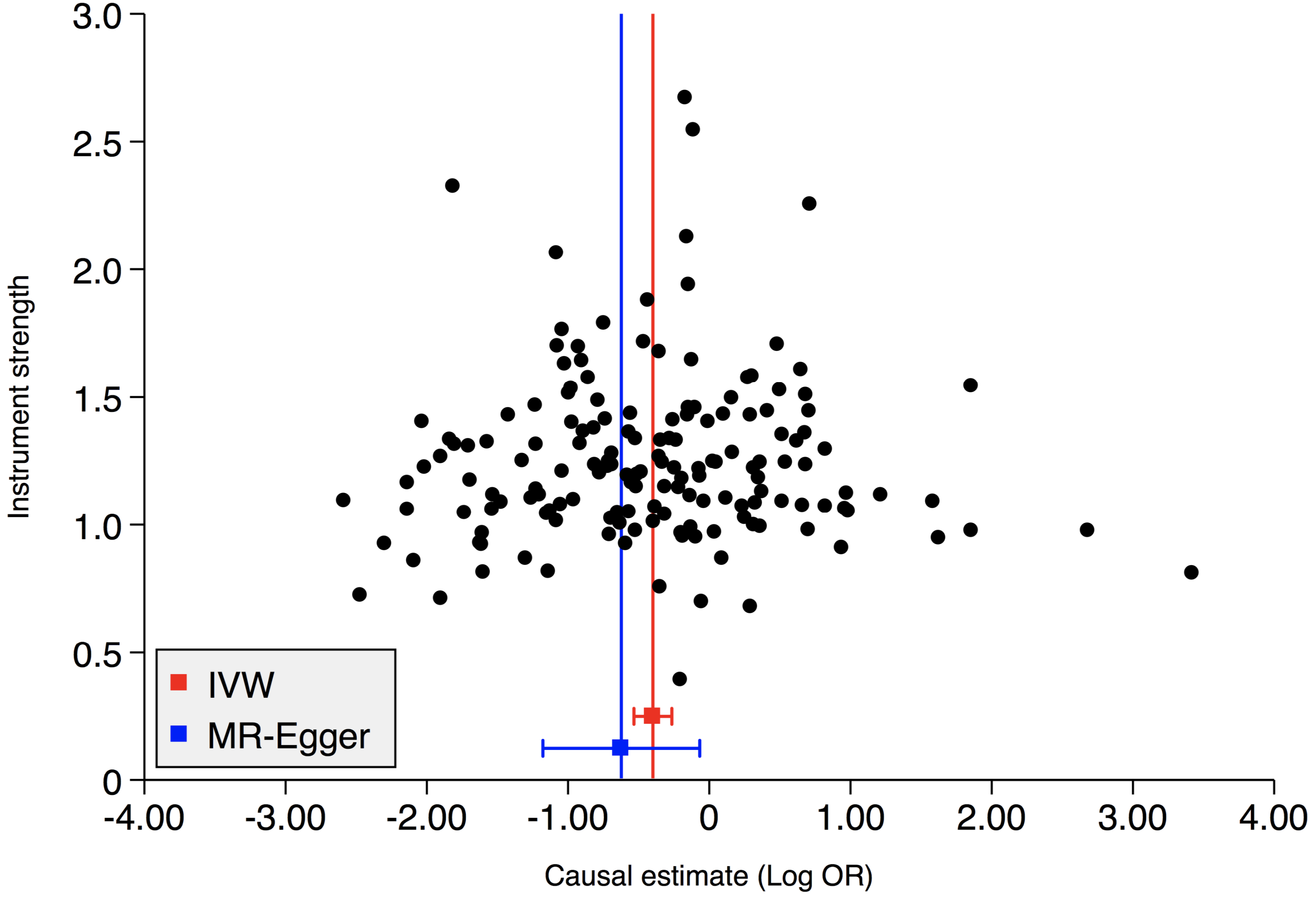


The instrument strength, representing the minor allele frequency corrected genetic association with education is calculated by dividing the SNP-exposure association by the standard error of the SNP-outcome association for each SNP. The conventional MR (IVW in red) and Egger MR (MR-Egger in blue) causal effect estimates are presented. SNP, single nucleotide polymorphism. CHD, coronary heart disease. IVW, inverse variance weighted approach. MR, Mendelian randomization. OR, odds ratio. CI, confidence interval.

**Supplementary Figure 15** Scatter plot of the 72 SNPs associated with education and their risk of CHD (with 95% confidence intervals)

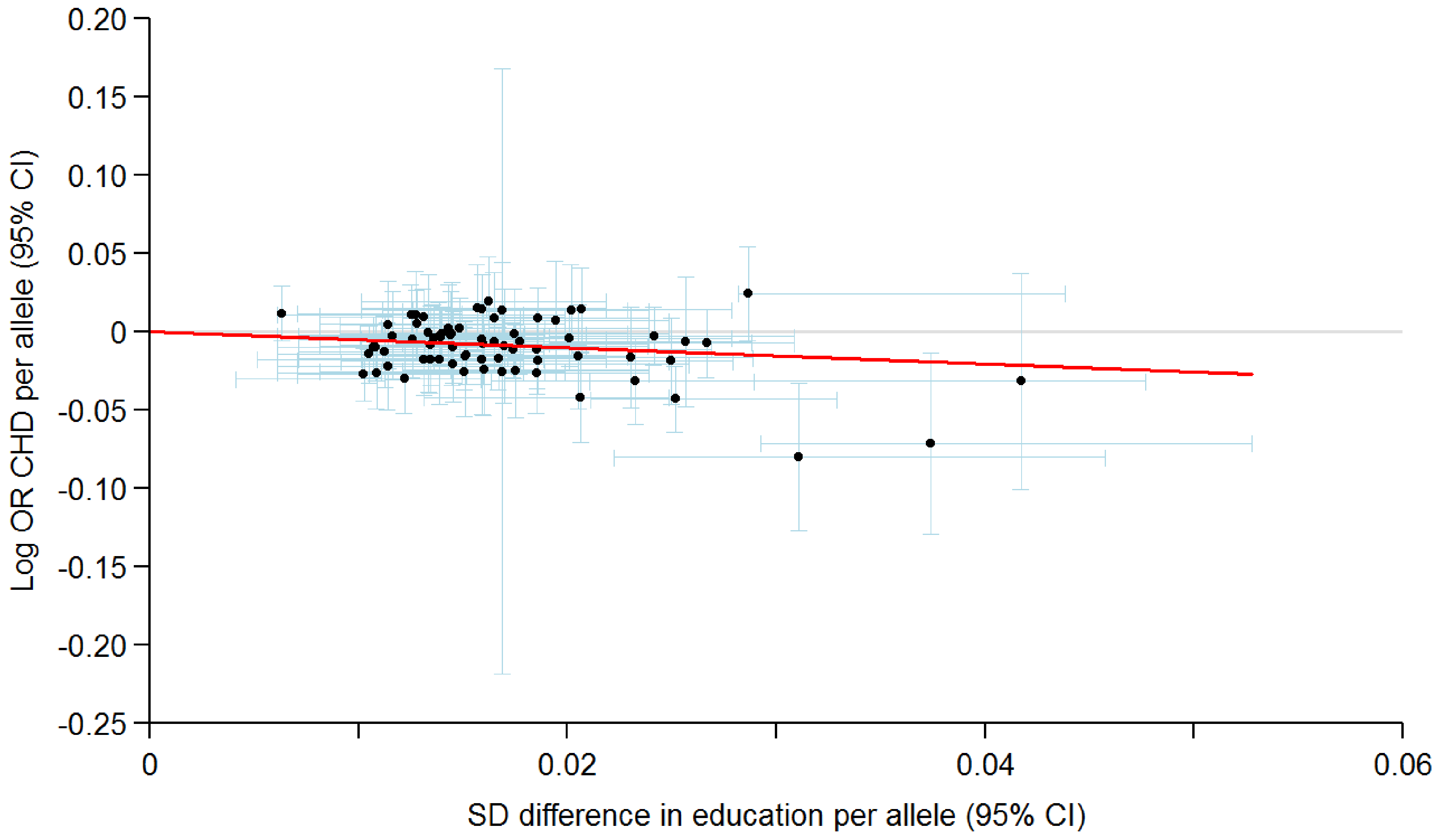


Each dot represents one single nucleotide polymorphism (SNP).
The red line represents the regression slope of the causal effect estimate of education on risk of CHD (where each SNP is weighted by its inverse allele frequency).
CHD, coronary heart disease. OR, odds ratio. CI, confidence interval.

**Supplementary Figure 16** Scatter plot of the 72 SNPs associated with education and their risk of CHD (with MR-Egger)

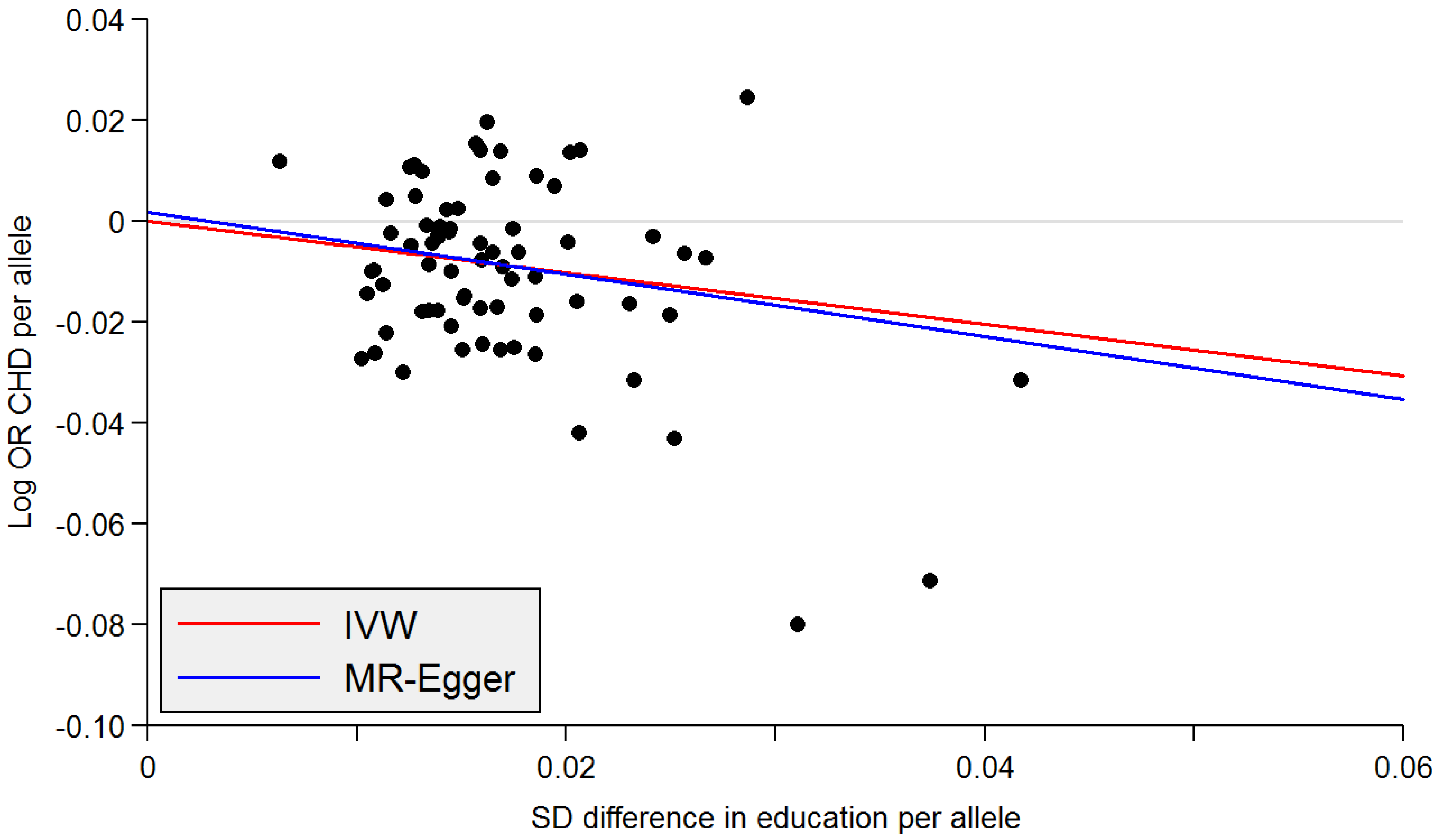


The red line shows causal regression estimates from conventional Mendelian randomization (MR), inverse variance weighted (IVW).
The blue line shows causal regression estimates from MR-Egger.
SNP, single nucleotide polymorphism. CHD, coronary heart disease. OR, odds ratio.

**Supplementary Figure 17** Funnel plot of 72 SNPs, showing instrument strength against causal estimates

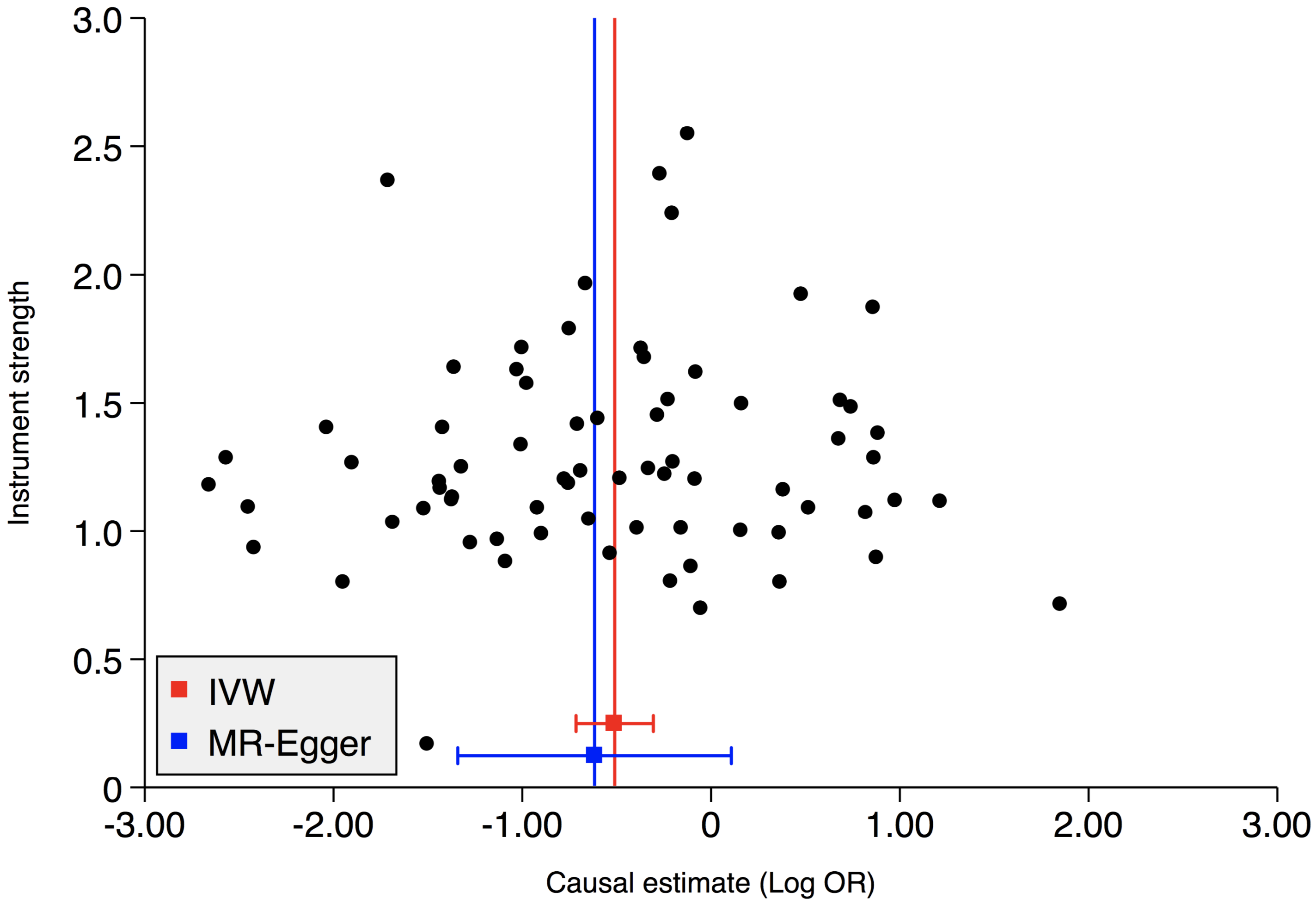


The instrument strength, representing the minor allele frequency corrected genetic association with education is calculated by dividing the SNP-exposure association by the standard error of the SNP-outcome association for each SNP. The conventional MR (IVW in red) and Egger MR (MR-Egger in blue) causal effect estimates are presented. SNP, single nucleotide polymorphism. CHD, coronary heart disease. IVW, inverse variance weighted approach. MR, Mendelian randomization. OR, odds ratio. CI, confidence interval.

**Supplementary Figure 18** Scatter plot of the 53 SNPs associated with CHD development and their educational outcomes (with 95% confidence intervals)

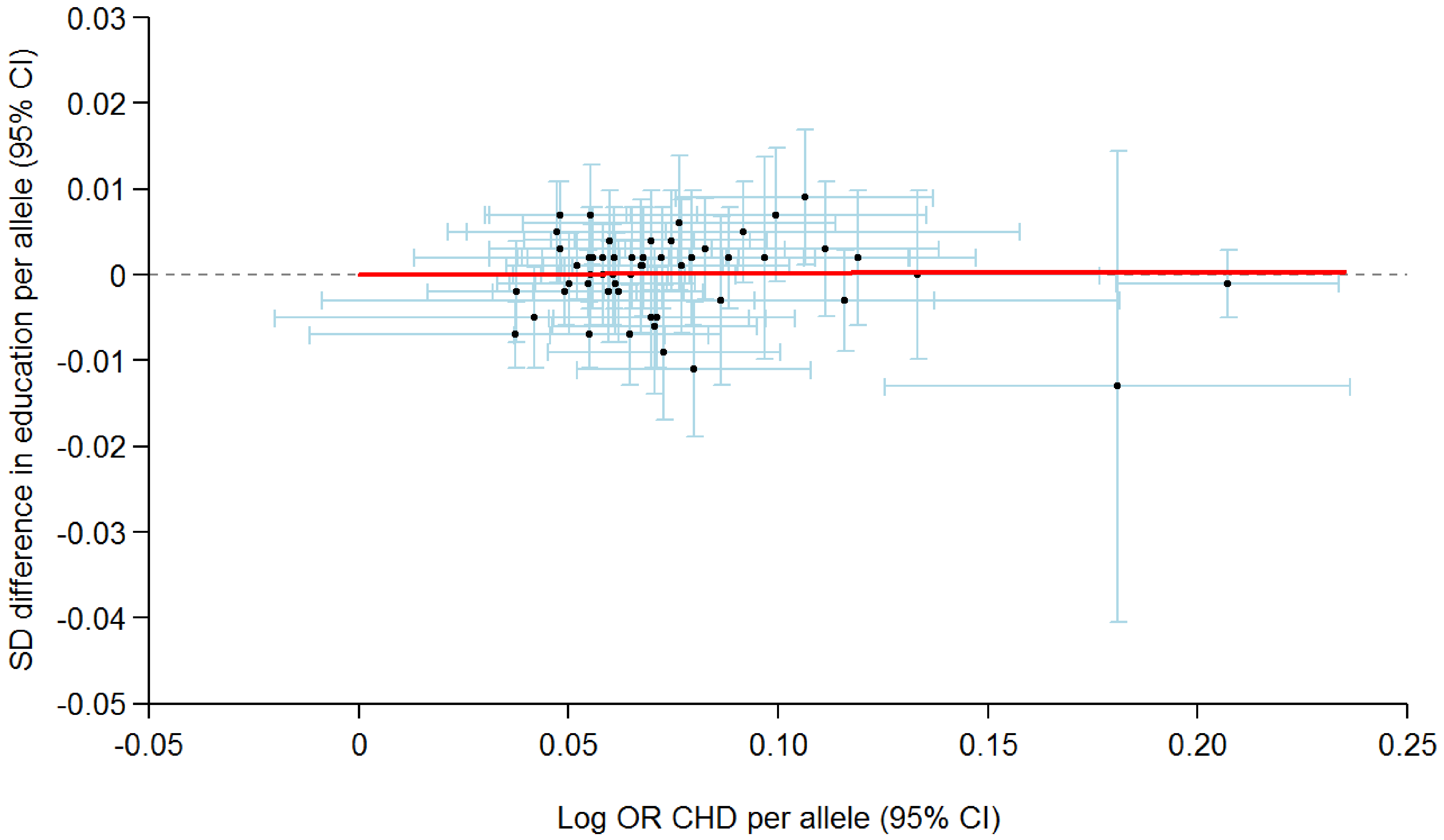


The red line represents the regression slope of the causal effects estimates (derived by the inverse-variance weighted Mendelian randomization method).
SNP, single nucleotide polymorphism. CHD, coronary heart disease. OR, odds ratio. CI, confidence interval.

**Supplementary Figure 19** Scatter plot of the 53 SNPs associated with CHD development and their educational outcomes (with MR-Egger)

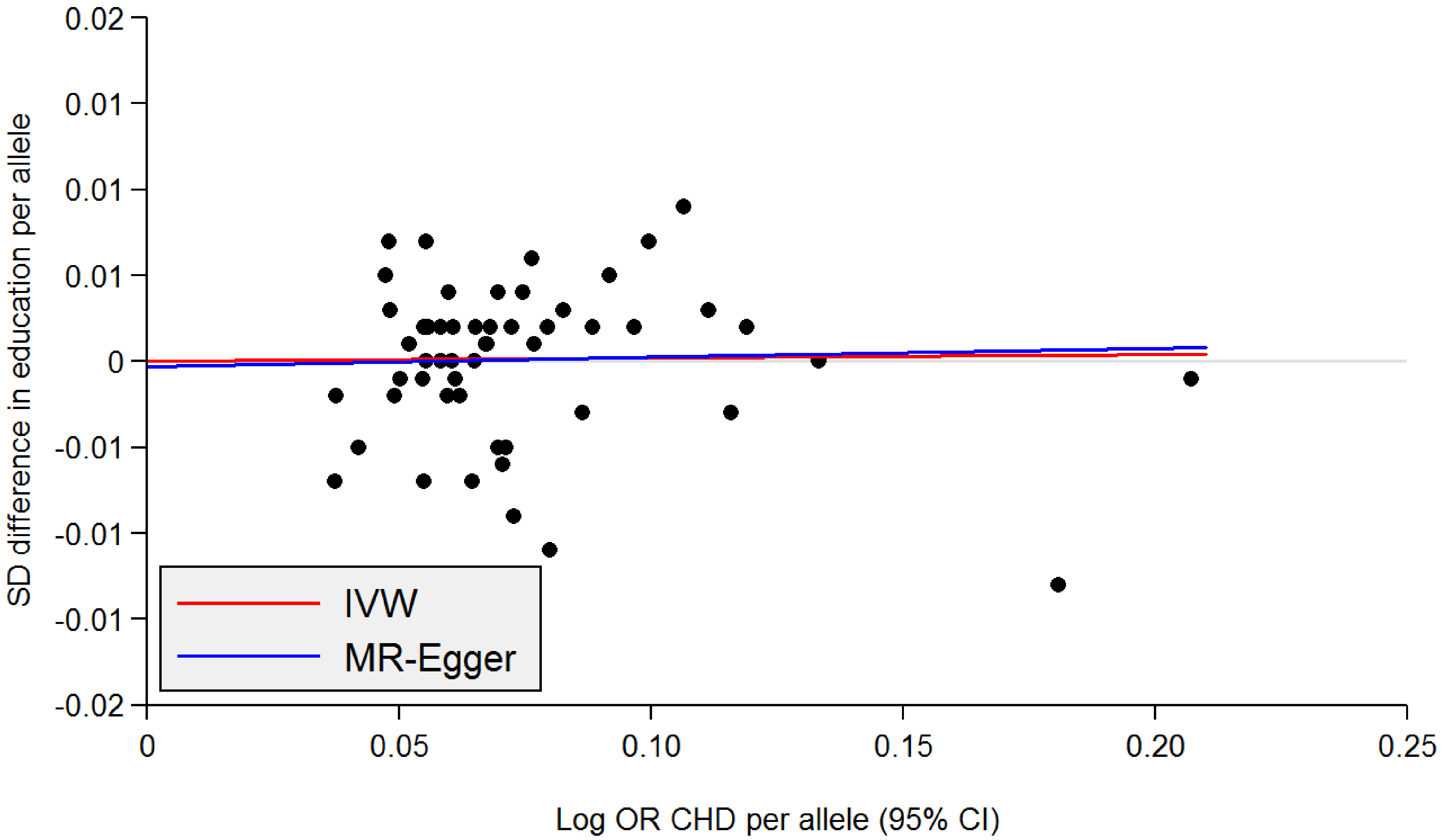


The red line shows causal regression estimates from conventional Mendelian randomization (MR), inverse variance weighted (IVW).

The blue line shows causal regression estimates from MR-Egger. Here, the MR-Egger intercept is -0.00027 (95% CI =-0.0029 to 0.0023) and Egger test p-value = 0.837, suggesting limited pleiotropy.

SNP, single nucleotide polymorphism. CHD, coronary heart disease. OR, odds ratio.

**Supplementary Figure 20** Funnel plot of 53 SNPs, showing instrument strength against the causal estimates (genetic liability for CHD on educational outcomes)

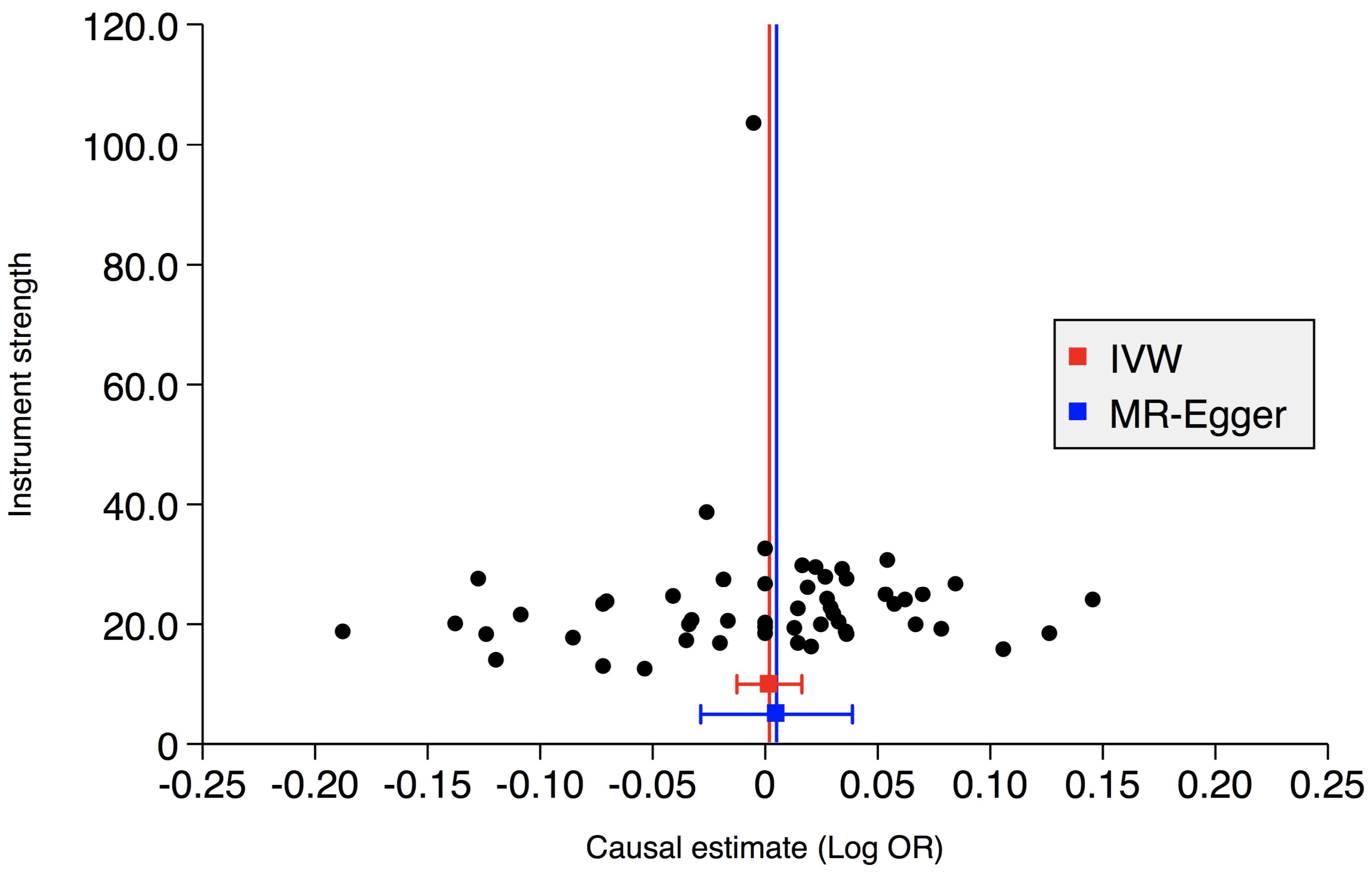


The instrument strength, representing the minor allele frequency corrected genetic association with risk of CHD is calculated by dividing the SNP-exposure association by the standard error of the SNP-outcome association for each SNP. The conventional MR (IVW in red) and Egger MR (MR-Egger in blue) causal effect estimates are presented. SNP, single nucleotide polymorphism. CHD, coronary heart disease. IVW, inverse variance weighted approach. MR, Mendelian randomization. OR, odds ratio. CI, confidence interval.

## Supplementary references


1. Kitagawa EM, Hauser PM. Differential mortality in the United States: A study in socio-economic epidemiology. 1st Edition. Cambridge, Mass: Harvard University Press; 1973.
2. Peasey A, Bobak M, Kubinova R, Malyutina S, Pajak A, Tamosiunas A, et al. Determinants of cardiovascular disease and other non-communicable diseases in Central and Eastern Europe: rationale and design of the HAPIEE study. *BMC Public Health* 2006;6:255.
3. Evans A, Salomaa V, Kulathinal S, Asplund K, Cambien F, Ferrario M, et al. MORGAM (an international pooling of cardiovascular cohorts). *Int J Epidemiol* 2005;34:21-7.
4. Ferrario MM, Veronesi G, Chambless LE, Tunstall-Pedoe H, Kuulasmaa K, Salomaa V, et al. The contribution of educational class in improving accuracy of cardiovascular risk prediction across European regions: The MORGAM Project Cohort Component. *Heart* 2014;100:1179-87.
5. Zheng J, Erzurumluoglu AM, Elsworth BL, Kemp JP, Howe L, Haycock PC, et al. LD Hub: a centralized database and web interface to perform LD score regression that maximizes the potential of summary level GWAS data for SNP heritability and genetic correlation analysis. *Bioinformatics* 2017;33:272-79.
6. Burgess S, Davies NM, Thompson SG. Bias due to participant overlap in two-sample Mendelian randomization. *Genet Epidemiol* 2016;40:597-608.
7. Okbay A, Beauchamp JP, Fontana MA, Lee JJ, Pers TH, Rietveld CA, et al. Genome-wide association study identifies 74 loci associated with educational attainment. *Nature* 2016;533:539-42.
8. Johnson AD, Handsaker RE, Pulit SL, Nizzari MM, O'Donnell CJ, de Bakker PI. SNAP: a web-based tool for identification and annotation of proxy SNPs using HapMap. *Bioinformatics* 2008;24:2938-9. (http://www.broadinstitute.org/mpg/snap/ldsearch.php).
9. Bowden J, Davey Smith G, Burgess S. Mendelian randomization with invalid instruments: effect estimation and bias detection through Egger regression. *Int J Epidemiol* 2015;44:512-25.
10. Bowden J, Davey Smith G, Haycock PC, Burgess S. Consistent Estimation in Mendelian Randomization with Some Invalid Instruments Using a Weighted Median Estimator. *Genet Epidemiol* 2016;40:304-14.
11. Bowden J, Del Greco MF, Minelli C, Davey Smith G, Sheehan NA, Thompson JR. Assessing the suitability of summary data for two-sample Mendelian randomization analyses using MR-Egger regression: the role of the I2 statistic. *Int J Epidemiol* 2016;45:1961-74.
12. Greco M FD, Minelli C, Sheehan NA, Thompson JR. Detecting pleiotropy in Mendelian randomisation studies with summary data and a continuous outcome. *Stat Med* 2015;34:2926-40.
13. Deloukas P, Kanoni S, Willenborg C, Farrall M, Assimes TL, Thompson JR, et al. Large-scale association analysis identifies new risk loci for coronary artery disease. *Nat Genet* 2013;45:25-33.
14. Schunkert H, Konig IR, Kathiresan S, Reilly MP, Assimes TL, Holm H, et al. Large-scale association analysis identifies 13 new susceptibility loci for coronary artery disease. *Nat Genet* 2011;43:333-8.
15. A genome-wide association study in Europeans and South Asians identifies five new loci for coronary artery disease. *Nat Genet* 2011;43:339-44.
16. Nikpay M, Goel A, Won HH, Hall LM, Willenborg C, Kanoni S, et al. A comprehensive 1,000 Genomes-based genome-wide association meta-analysis of coronary artery disease. *Nat Genet* 2015;47:1121-30.
17. Burgess S, Thompson SG. Multivariable Mendelian randomization: the use of pleiotropic genetic variants to estimate causal effects. *Am J Epidemiol* 2015;181:251-60.
18. Genome-wide meta-analyses identify multiple loci associated with smoking behavior. Nature genetics. 2010;42(5):441-7.
19. Wain LV, Verwoert GC, O'Reilly PF, Shi G, Johnson T, Johnson AD, et al. Genome-wide association study identifies six new loci influencing pulse pressure and mean arterial pressure. *Nat Genet* 2011;43:1005-11.
20. Willer CJ, Schmidt EM, Sengupta S, Peloso GM, Gustafsson S, Kanoni S, et al. Discovery and refinement of loci associated with lipid levels. *Nat Genet* 2013;45:1274-83.
21. Morris AP, Voight BF, Teslovich TM, Ferreira T, Segre AV, Steinthorsdottir V, et al. Large-scale association analysis provides insights into the genetic architecture and pathophysiology of type 2 diabetes. *Nat Genet* 2012;44:981-90.
22. Dupuis J, Langenberg C, Prokopenko I, Saxena R, Soranzo N, Jackson AU, et al. New genetic loci implicated in fasting glucose homeostasis and their impact on type 2 diabetes risk. *Nat Genet* 2010;42:105-16.
23. Wood AR, Esko T, Yang J, Vedantam S, Pers TH, Gustafsson S, et al. Defining the role of common variation in the genomic and biological architecture of adult human height. *Nat Genet* 2014;46:1173-86.
24. Locke AE, Kahali B, Berndt SI, Justice AE, Pers TH, Day FR, et al. Genetic studies of body mass index yield new insights for obesity biology. *Nature* 2015;518:197-206.
25. Brion MJ, Shakhbazov K, Visscher PM. Calculating statistical power in Mendelian randomization studies. *Int J Epidemiol* 2013;42:1497-501.
26. White J, Swerdlow DI, Preiss D, et al. Association of lipid fractions with risks for coronary artery disease and diabetes. *JAMA Cardiol* 2016;1:692-9.
